# Supplementary material for: Complex trait susceptibilities and population diversity in a sample of 4,145 Russians
Source: Nat Commun. 2024 Jul 23;15:6212. doi: 10.1038/s41467-024-50304-1 (PMC11266540; doi:10.1038/s41467-024-50304-1)
Supplement: Supplementary file 1 — Supplementary Information [file 41467_2024_50304_MOESM1_ESM.pdf]

Understanding Complex Trait Susceptibilities and Population Diversity in a Sample of 4,145 Russians Through Analysis of Clinical and Genetic Data

Dmitrii Usoltsev[1-5], Nikita Kolosov[1-5], Oxana Rotar[1], Alexander Loboda[1-3], Maria Boyarinova[1], Ekaterina Moguchaya[1], Ekaterina Kolesova[1], Anastasia Erina[1], Kristina Tolkunova[1], Valeriia Rezapova[1-3], Ivan Molotkov[4,5], Olesya Melnik[1], Olga Freylikhman[1], Nadezhda Paskar[1], Asiiat Alieva[1], Elena Baranova[1], Elena Bazhenova[1], Olga Beliaeva[1], Elena Vasilyeva[1], Sofia Kibkalo[1], Rostislav Skitchenko[1], Alina Babenko[1], Alexey Sergushichev[2], Alena Dushina[6], Ekaterina Lopina[6], Irina Basyrova[6], Roman Libis[6], Dmitrii Duplyakov[7,8], Natalya Cherepanova[8], Kati Donner[9], Paivi Laiho[10], Anna Kostareva[1-2], Alexandra Konradi[1-2], Evgeny Shlyakhto[1], Aarno Palotie[3,9,11], Mark J. Daly[3,9,11] and Mykyta Artomov[1-5,9,11]

1 - Almazov National Medical Research Centre, St. Petersburg, Russia
2 - ITMO University, St. Petersburg, Russia
3 - Broad Institute, Cambridge, MA, USA
4 - The Institute for Genomic Medicine, Nationwide Children's Hospital, Columbus, USA
5 - Department of Pediatrics, The Ohio State University College of Medicine, Columbus, OH, USA
6 - Orenburg State Medical University, Orenburg, Russia
7 - Samara State Medical University, Samara, Russia
8 - Samara Regional Cardiology Dispensary, Samara, Russia
9 - Institute for Molecular Medicine Finland (FIMM), Helsinki, Finland
10 - Finnish Institute for Health and Welfare (THL), Helsinki, Finland
11 - Analytic and Translational Genetics Unit, Massachusetts General Hospital, Boston, MA, USA

Correspondence: mykyta.artomov@nationwidechildrens.org

Supplementary Notes

Table of contents

Supplementary Note 1 ESSE Data Collection 3

- **Supplementary Note 1.1 Questionnaire** 3
- **Supplementary Note 1.2 Physical Examination** 6
- **Supplementary Note 1.3 Blood biomarkers** 7
- **Supplementary Note 1.4 Vascular assessment** 8
- **Supplementary Note 1.5 CVD risk scales** 9

Supplementary Note 2 ESSE Follow-up Data Collection 9

Supplementary Note 3 Starvation Study Controls Data Collection 9

Supplementary Note 4 Genetic data generation and quality control 10

- **Supplementary Note 4.1 Genotype imputation** 10
- **Supplementary Note 4.2 Validation of systematic sex-mismatch absence** 11
- **Supplementary Note 4.3 Kinship analysis** 13
- **Supplementary Note 4.4 Genetic Data Quality Control** 14

Supplementary Note 5 Principal Component Analysis 15

Supplementary Note 6 Comparison of Russian samples with 1000 Genomes 17

Supplementary Note 7 Clustering of Russian samples 18

Supplementary Note 8 Admixture analysis 18

Supplementary Note 9 Fst estimation 20

Supplementary Note 10 Identity-by-descent (IBD) estimation 22

Supplementary Note 11 Population analysis utilizing only genotyped variants 24

Supplementary Note 12 Enrichment of Finnish and Russian variants 25

Supplementary Note 13 Estimated population size 28

Supplementary Note 14 Population Tree Construction 28

Supplementary Note 15 GWAS 31

Supplementary Note 1 ESSE Data Collection

A national study "Epidemiology of cardiovascular diseases in different regions of the Russian Federation" (ESSE-RF) was launched in 2012—2013 in 12 regions of Russia, different in climatic, geographic, economic and demographic characteristics. Within the framework of this study, a stratified multistage random sample of 4,800 residents (the adult population - men and women aged 46 ± 12 SD years) was formed in St. Petersburg, Orenburg and Samara. All participants signed informed consent and completed a standard questionnaire based on adapted and validated international methods.

Respondents were invited for a one-day ambulatory visit. First, they filled out a questionnaire, then they underwent anthropometry and donated blood samples. In St. Petersburg, participants also underwent electrocardiogram (ECG) tests and detailed assessments of their vascular health, along with advanced blood tests.

Supplementary Note 1.1 Questionnaire

The questionnaire consisted of 10 sections. The first section collected common information about the respondent, including contact details, the date of the ambulatory visit, age, sex, birthdate, family status (never married / currently married / divorced / widowed), education level (primary / incomplete secondary / complete secondary / professional with complete secondary / special secondary / incomplete higher / higher), employment status (currently employed / never worked / unemployed / retired / disabled), and whether the respondent had children (yes, with the number of children / no).

The second section of the questionnaire focused on dietary behaviors. It included questions about:

- Salt consumption habits (always add salt to food / add salt to food before tasting / don't add salt to food).
- Types of fat used for cooking (vegetable oil / margarine / butter / animal fat / no fat at all / mixture of vegetable and animal fats).
- Types of fat used for spreading on bread and/or adding to cereals (none / soft margarine / solid margarine / butter / lard / mixture of vegetable and animal fats).
- Number of lumps and/or teaspoons of sugar (honey, jam, etc.) consumed per day.
- Frequency of consuming red meat, fish or seafood, poultry meat, pickled foods, sausages, pasta or cereals, fresh vegetables and fruits, legumes, sweets and confectionery, milk or kefir or yogurt, sour cream, cottage cheese, and cheese (options: I don't use or rarely / 1-2 times per month / 1-2 times per week / daily or almost daily).
- Fat content of kefir milk, yogurt, sour cream, cottage cheese, and cheese used.

High salt levels were considered if participants added salt to food or consumed pickled foods daily. High sugar consumption was considered if participants consumed ≥ 6 lumps and/or teaspoons of sugar (honey, jam, etc.) per day or consumed sweets daily. High fat consumption was considered if participants used butter/animal fat for cooking or used butter/lard for

spreading on bread and/or adding to cereals. Insufficient consumption of fresh vegetables and fruits was considered if consumption was less than daily. Insufficient fish consumption was considered if consumption was less than 1-2 times per week.

The third section focused on the physical activity of respondents and included four questions:

- "Which of the following levels of physical activity best describes your physical load during work?" (Options: Mostly sit / mostly walk / lifting and carrying weights / do hard physical work / don’t work).
- "How many minutes per day do you walk outside of work, including walking to and from your place of work?"
- "How many times a week, in your free time, do you engage in hard physical activity lasting at least 20-30 minutes, such that you experience a little shortness of breath or sweat?"
- "On average, how much time did you spend sitting on a weekday in the last week?"

Sufficient physical activity was considered as walking for ≥ 150 minutes per day and engaging in hard physical activity for ≥ 3 times per week. Low physical activity was considered as walking for < 150 minutes per day.

The fourth section covered smoking habits and included questions about:

- Smoking status (never smoked / smoked but quit / current smoker).
- Daily smoking (yes / no).
- Age of smoking initiation.
- Smoking quitting and number of cigarettes per day.

The smoking index was calculated as the number of cigarettes per day multiplied by the smoking history (in age) divided by 20. A smoking index ≥ 10 was considered risky. We also defined a group of current smokers additionally including those who had quit smoking for less than a year.

The fifth section asked about how often alcohol was consumed (beer, dry wine/champagne, fortified wine, homemade tinctures, spirits), the usual amount consumed at one time, and the amount consumed in the last week. This section also included the Alcohol Use Disorders Identification Test (AUDIT). Excessive alcohol consumption was considered as ≥ 168g of pure alcohol per week for males and ≥ 84g for females. We also identified a group of non-drinkers.

We categorized individuals based on their disorder status (obesity or arterial hypertension) and several lifestyle habits (high salt, sugar, fat, alcohol consumption, smoking, and low physical activity) for each pair of phenotypes. If an individual did not have a disorder or an unhealthy habit, they were included in group 0. If an individual had a disorder but no

unhealthy habit, they were included in group 1. If an individual did not have a disorder but had an unhealthy habit, they were included in group 2. If an individual had both a disorder and an unhealthy habit, they were included in group 3. Additionally, we created a binary factor to compare group 3 with the others (0, 1, 2).

The sixth section included questions to assess self-perceived health:

- How do you rate your current health? (Scale from 1 to 5).
- Compared to others your age, how do you rate your health? (Better, same, worse).

This section also collected information on menstruation history, pregnancy, and birth weight of the child:

- Do you menstruate?
- Age of last menstruation.
- Reason for menstrual cessation (age, surgical menopause with ovary removal, surgical menopause without ovary removal).
- Hormone replacement therapy (yes, no) and hormonal contraception (yes, no).
- Number of pregnancies/births and birth weight of the child.
- Experience of late fetal loss (after 22 weeks of pregnancy).
- Preterm birth history (before 37 weeks of pregnancy).
- History of increased blood pressure in the second half of pregnancy.

The seventh section focused on sleep issues and included 7 questions:

- What was your average daily sleep duration in the past month?
- How often did you find it difficult to fall asleep within 30 minutes after going to bed?
- How often did you find it difficult to fall back asleep after waking up in the middle of the night?
- How often did you find it difficult to stay awake when you needed to?
- How often did you need medication to help you fall asleep?
- Do you snore while sleeping?
- Do you experience respiratory arrests during sleep?

The eighth section covered health history with questions such as:

- Are you aware of your blood pressure and cholesterol levels?
- Has your doctor informed you about high blood pressure, glucose, or cholesterol levels?
- Have you taken any medications for blood pressure, sugar, or lipid lowering in the past 2 weeks?
- Do you experience regular headaches, and have you consulted a doctor about them? If so, what was the diagnosis (hypertension, migraine, or other)?
- Have you ever experienced sudden, short-term weakness or numbness in one arm, leg, or side of your face?
- Have you had sudden, short-term vision loss in one eye?
- Have you experienced sudden dizziness or instability while walking?
- Have you been diagnosed with a hypertensive crisis?
- Have you ever been treated with glucocorticoids or anticonvulsants?
- Have you been diagnosed with or treated for conditions such as osteochondrosis, spondylitis, rheumatoid arthritis, asthma, chronic bronchitis, stroke, myocardial infarction, ischemic heart disease, heart rhythm disorders, liver or gallbladder

diseases, gastrointestinal tract disorders, stomach or duodenal ulcers, kidney diseases, thyroid diseases, cancer, organ transplants, or diabetes mellitus (any type)?
- We also asked about the family history of myocardial infarction, stroke, arterial hypertension, diabetes mellitus, and hip fractures.

The ninth section of the questionnaire focused on the economic conditions of patients with the following questions:
- What are the sources of income in your family?
- What portion of your income is typically spent on food?
- Please choose the statement that best describes your family's financial situation:
  We don't even have enough for basic necessities.
  We can buy everything we need but not expensive durable goods.
  From time to time, we can purchase expensive durable goods.
  We can buy durable goods but not things like an apartment, a house, or an expensive car.
  We are able to buy things like a house, an apartment, or an expensive car.
- How do you rate your family's well-being compared to others?
  Very wealthy
  Relatively wealthy
  Average
  Relatively poor
  Very poor

The final section comprises a questionnaire evaluating perceived stress (PSS) [1] and the assessment of depression and anxiety was conducted using the Hospital Anxiety and Depression Scale (HADS) [2].

The Rose questionnaire was used as a screening test for the diagnosis of Angina pectoris [3,4]. The standard questionnaire consisted of 9 questions that identify predictors of angina. Questions 10, 11 and 12 were added by the Federal State Budgetary Institution National Medical Research Center for Therapy and Preventive Medicine (department of multifactorial prophylaxis). Additionally, intermittent claudication and risk of heart attack were assessed.

Supplementary Note 1.2 Physical Examination

All participants underwent anthropometric measurements. Body weight was measured using VEM-150- Mass-K scales (Russia), height was measured with the RM-1 Diacoms height meter (Russia), and waist and hip circumferences were measured using a standard flexible centimeter tape. Abdominal obesity was determined according to the JIS 2009 metabolic syndrome criteria [5] as: waist circumference (WC) ≥94 cm for males and ≥80 cm for females. Additional less strict criterion was ≥102 cm for males and ≥88 cm for females. Obesity based on waist to hip circumference ratio was defined as ≥0.9 for males and ≥0.85 for females. Body mass index (BMI) was calculated using the Quetelet formula as the ratio of body weight in kilograms to height in meters squared. All respondents were classified as obese (BMI ≥ 30

kg/m2), overweight (25 kg/m2 ≤ BMI <30 kg/m2), normal weight (18.5 kg/m2 ≤ BMI < 25 kg/m2) and underweight (BMI < 18.5 kg/m2). Additionally, obese group (BMI ≥ 30 kg/m2) was separated into 3 subgroups according to the degree of obesity: 1 degree (30 kg/m2 ≤ BMI <35 kg/m2), 2 degree (35 kg/m2 ≤ BMI <40 kg/m2), 3 degree (≥40 kg/m2). Finally, we defined groups of individuals who had at least 1 type of obesity (abdominal, waist to hip circumference ratio or BMI).

Blood pressure (BP) and heart rate (HR) were measured by the OMRON tonometer (Japan) after resting for 5 minutes in a sitting position twice on the right (N=4,339) or left (N=457) hand with intervals of 2 minutes. The average BP and HR of the two measurements was calculated. Antihypertensive treatment in the past 2 weeks was clarified through a questionnaire. Two cutt-off levels were applied to BP: ≥ 140/90 mmHg according to hypertension guidelines [6] and ≥ 135/80 mmHg according to metabolic syndrome consensus [5]. All responders classified as having arterial hypertension (AH) (BP ≥ 140/90 mmHg or antihypertensive treatment), high-normal BP (135/85 mmHg ≤ BP <140/90 mmHg, no antihypertensive treatment) normal BP (120/80 mmHg ≤ BP <135/85 mmHg, no antihypertensive treatment) and optimal BP (BP < 120/80 mmHg, no antihypertensive treatment). High-normal BP and normal BP was considered as prehypertension. Effective antihypertensive treatment was defined as achieving BP below 140/90 mmHg, with stricter criteria indicating BP below 130/80 mmHg [6].

We calculated the Estimated Pulse Wave velocity as it was defined previously [7]. Mean blood pressure was calculated as DBP + 0.4*(SBP - DBP). Responders from St. Petersburg additionally had orthostatic BP and heart rate measurement after 3 minutes in standing position.

Electrocardiogram (ECG) registration was carried out using a computerized PADSY complex (Medset Medizintechnik GmbH, Germany), interpretation was carried out according to a standard protocol.

Supplementary Note 1.3 Blood biomarkers

3 areas (St. Petersburg, Samara, Orenburg)

The blood glucose (mmol/L), creatinine (μmol/L), uric acid (μmol/L.), and lipids (mmol/L) measurement (total cholesterol – TC, low-density lipoproteins – LDL, high-density lipoproteins – HDL, triglycerides – TG) were performed in fasting state (Abbott Architect 8000, USA; reagents Abbott Diagnostic). We defined several groups of respondents according to glucose levels: 1st group (glucose < 5.6 mmol/L), 2nd group (5.6 mmol/L ≤ glucose < 7

mmol/L), 3rd group (7 mmol/L ≤ glucose < 11 mmol/L) and 4th group (glucose ≥ 11 mmol/L). Glucose lowering and lipid lowering therapy in the past 2 weeks were clarified through a questionnaire. Dyslipidemia was considered for individuals with increased cholesterol > 4.9 mmol/L or LDL > 3 mmol/L or TG > 1.7 mmol/L or reduced HDL (in male <1.0 and in female <1.2 mmol/L) or presence of lipid-lowering therapy [8]. Additionally, the atherogenic index of plasma (AIP) was calculated as logarithmic ratio between triglycerides and HDL: $\log_2(TG/HDL)$. The glomerular filtration rate (GFR) was calculated using the CKD-EPI formula [9]. Diabetes was diagnosed in case of anamnesis (information from patient).

Insulin (pmol/L) and N-terminal pro-hormone of brain natriuretic peptide (pro-BNP, pg/ml) levels were performed in fasting state (Cobas Integra 400 plus, Switzerland; Roche-diagnostics reagents). Two cutt-off levels were applied to pro-BNP (pro-BNP >125 pg/ml and pro-BNP > 300 pg/mL) [10]. Insulin resistance was evaluated using the Homeostasis Model Assessment of Insulin Resistance (HOMA-IR) index using the formula: fasting blood glucose × insulin × 0.138 (coefficient which is used to convert pmol/L to μU/ml) / 22.5. Insulin resistance was defined in the case of HOMA-IR ≥ 2.6. The triglyceride glucose (TyG) index was calculated as a natural logarithm: ln(TG[mmol/L] × 87.5 × glucose[mmol/L] × 18/2). Modifications of the TyG index were calculated as TyG_BMI = TyG × BMI, TyG_WC = TyG × Waist circumference and TyG_WC_HEI = TyG × Waist circumference/Height [11]. Low insulin levels were considered as < 17.8 pmol/L, high as > 173 pmol/L. The FINDRISC scale was used to assess risks of diabetes [12].

Additionally, the metabolic syndrome was determined according to the following criteria (presence of three or more components: SBP ≥130 or DBP ≥85 mm Hg or antihypertensive therapy; triglycerides ≥1.70 mmol/L; HDL <1.04 (males)/1.30 (females) mmol/L or lipid-lowering therapy; glucose ≥5.6 mmol/L or hypoglycemic therapy; WC >102 (males)/88 (females)) in combination with the absence of cardiovascular diseases (CVD) and diabetes mellitus (DM) at the time of inclusion in the study, according to the anamnesis [13].

St. Petersburg only (additional blood biomarkers)

The leptin and adiponectin levels were determined by enzyme-like immunoassay (DRG, Germany). C-reactive protein (CRP,mg/L), thyroid-stimulating hormone (TSH,mIU/L) and cortisol (μmol/L) were measured in fasting blood samples (Cobas Integra 400 plus, Switzerland; Roche-diagnostics reagents). Two cut-off levels of CRP were applied: CRP > 3 mg/L and CRP > 1 mg/L [14]. TSH > 4 mIU/L was defined as a high TSH level [15,16] Low TSH was considered as < 0.4 mIU/L [15]. Low cortisol was defined as < 171 μmol/L.

Extended lipids study (lipoprotein (a), apolipoprotein A, apolipoprotein B) and urine microalbuminuria (albumin excretion in urine portion) was performed using (Abbott Architect 8000, USA; reagents Abbott Diagnostic). High lipoprotein (a) was considered as > 0.3 g/L [17]. Low apolipoprotein A was defined as <1.04 g/L for male and <1.08 g/L for female. High

apolipoprotein A was defined as >2.02 g/L for male and >2.25 g/L for female. Low apolipoprotein B was defined as <0.66 g/L for male and <0.6 g/L for female. High apolipoprotein B was defined as >1.33 g/L for male and >1.17 g/L for female. High urine albumin excretion was defined as >30 mg/dL [6].

Supplementary Note 1.4 Vascular assessment

Vascular assessment was performed only for St. Petersburg residents.

Ultrasound examination of the common carotid arteries was performed using a portable diagnostic system My Sono U6 (Samsung, Korea). The standard protocol included measurements bilaterally at a distance of 1 cm from the bifurcation of the common carotid artery along its posterior wall in three positions (anterior, middle, and posterior longitudinal). The thickness of the intima-media complex (IMT) was defined as the distance between the first and second echogenic line of the located vessel. Subsequently, the mean IMT on both sides was calculated as the arithmetic mean of three measurements. In addition, the presence or absence of atherosclerotic plaques was assessed. Values greater than 0.9 mm and less than 1.3 mm were taken as an increased IMT. Local thickening ≥ 1.3 mm was regarded as atherosclerotic plaques [18].

The carotid-femoral pulse wave velocity (cfPWV) was assessed using a SphygmoCor device (AtCor, Australia). Carotid-femoral distance was measured using the formula recommended by the 2012 Expert Consensus on Vascular Stiffness: (distance from common carotid artery to common femoral artery in cm) × 0.8. Using a special sensor, the applanation method recorded pulse waves for 10 seconds, first in the projection of the common carotid artery on the left, then in the common femoral artery on the left, also for at least 10 seconds. An indicator of less than 10 m/s for PWV was taken as the normal value [19].

Cardio-ankle vascular index (CAVI) was measured automatically on the right and left using the VaSera VS-1500 device (Fukuda Denshi, Japan). CAVI was calculated between the heart valve and the ankle artery using the FCG signal (II tone) and plethysmographs obtained by applying cuffs to the upper arm and lower leg. The value of the CAVI > 9.0 was considered as elevated [20]. Measurement of ankle-brachial index (ABI) was also performed automatically on the right and left, calculated as the ratio of SBP on the leg (at the ankle) to SBP on the brachial bilaterally artery. ABI less than 0,9 at least one side was considered decreased [21].

Supplementary Note 1.5 CVD risk scales

Several cardiovascular risk scores were calculated using well-known scales. Framingham risk score 2008 was calculated using mean parameters values obtained from the ESSE cohort and from the original study [22], and using Framingham risk score 2008 tables. ASCVD risk estimator 2013 was calculated with mean parameter values from the ESSE cohort and from the original study [23]. SCORE systemes 2003, 2016 and 2019 were also calculated [24–26]. It's important to note that the SCORE system predicts only fatal cardiovascular disease. To convert fatal risk to total (non-fatal + fatal), the SCORE-High ASCVD risk results were

multiplied by 3 for men and by 4 for women [8]. The recalibration for the Russian population scale of SCORE 2016 - SCORE-MoSP was also calculated [27].

Supplementary Note 2 ESSE Follow-up Data Collection

Additionally, we collected data about patients' vital status, cardiovascular events and new disease onset every two years (**Supplementary Data 1**). Biannually from 2013 to 2021, participants and their relatives were contacted by phone and e-mail to collect information regarding fatal events (all-cause mortality) and non-fatal events (myocardial infarction, unstable angina, stroke). Patients and their relatives were invited to the clinic where information was verified using medical documentation and death certifications.

289 patients from St. Petersburg were invited for detailed follow-up data collection visits in 2018-2019 as part of different local studies (familial hypercholesterolemia, metabolic healthy obesity, early vascular aging). The same phenotypic information as the first visit was collected using analogous protocols and methods (**Supplementary Note 1**). For all continuous phenotypes, the difference between the measurements at the first and second visits was calculated as delta.

Supplementary Note 3 Starvation Study Controls Data Collection

138 individuals were recruited in 2017-2018 in St. Petersburg as controls for local study of early childhood starvation effects. Respondents were invited for a one-day ambulatory visit.

Initially they filled out a short version of the ESSE questionnaire with questions about family status, education, smoking status, antihypertensive / glucose lowering / lipids lowering therapy and concomitant diseases. Anthropometry, blood pressure and heart rate in sitting and standing positions were collected using the same methods as for ESSE respondents (**Supplementary Note 1**). Blood samples were collected and biobanked. Fasting glucose, creatinine, and lipids were measured (**Supplementary Note 1**).

Supplementary Note 4 Genetic data generation and quality control

4,723 individuals (4,594 ESSE + 129 Starvation Controls) were genotyped using a custom FinnGen Affymetrix Axiom array [28]. After the genotype imputation with BEAGLE 4.0 [29] using Haplotype Reference Consortium (HRC) data as a reference panel [30] the resulting dataset contained 623,249 genotyped variants and 10,454,514 imputed variants. We exclude 252 (224 ESSE and 28 Starvation Controls) samples with sex mismatch.

Supplementary Note 4.1 Genotype imputation

Target genotypes preprocessing

Initially, we excluded variants with a call rate exceeding 5% (n=2,615) and individuals who were batch samples, possessed duplicated IDs, or exhibited a missing rate more than 5% (N=125). Additionally, we eliminated all ambiguous contigs, retaining only autosomes and

the X chromosome. Further, we left-aligned and normalized indels to the human reference genome (hg19), checked for any dataset-specific REF/ALT flips using bcftools *norm* function and kept only biallelic, non-duplicate variants afterwards. Additionally, we excluded variants showing highly discordant allele frequencies compared with the imputation reference panel frequencies (logFC > 5 or logFC < -5 or AF difference > 0.10), following the recommendations from FIMM imputation protocol: https://dx.doi.org/10.17504/protocols.io.xbgfijw. Further, genotypes were pre-phased using BEAGLE, strand-checked and matched to the HRC reference panel [29]using *conform-gt.jar* tool provided by the BEAGLE team (https://tinyurl.com/u6ekd3sc). In the end, we kept 474,430 variants and 4,723 individuals for further SNP imputation.

HRC reference panel preprocessing

We utilized the Haplotype Reference Consortium (HRC) whole-genome sequencing dataset as a reference panel for imputing genotypes of the studied cohort. Superiority of the aforementioned reference panel over other publicly available alternatives was shown elsewhere [30].

All variants were processed the same way as it was described in Kolosov et al. Additionally, we split the dataset into individual chromosomes and converted each *.vcf* to *.bref* (binary reference format) using *bref.*.jar* tools provided by the BEAGLE team to speed up SNP imputation. As a result, we kept 37,620,210 variants and 27,165 individuals for further analysis.

Imputation using Beagle

Imputation procedures were performed using Beagle 5.2 [31] with the default parameters (*burnin* = 6, *iterations* = 12, *imp-segment* = 6, *ne* = 1000000). The imputation quality for each variant was measured using Dosage-R2 (DR2), as given in Beagle output. All variants with DR2 > = 0.8 were considered well-imputed and kept for further analysis.

Imputation quality

We measured imputation quality for the Russian cohort using the “masking” approach, described previously [30]. We masked 5% (n=23,704) of directly genotyped variants from the initial dataset and put them aside until completion of imputation. We sampled them chromosome-wise to account for non-uniform distribution of variants between chromosomes. Afterwards, we compared genotypes for these variants in genotyped and imputed datasets to calculate imputation quality scores (IQS) and concordance of produced allele frequencies.

Imputation quality scores (IQS) were more than 0.8 for the majority of masking variants (74%; n=17,541), slight decrease in median IQS value were observed only for less frequent variants (MAF < 0.05), however, still exceeding considerable high values (IQS(median)=0.86) (**Sup. Fig. 1a**). Additionally, only 720 (3%) out of 23,704 were classified as discordant in terms of imputed allele frequency (logFC > 5 or logFC < -5 or AF difference>0.10) (**Sup. Fig. 1b**).

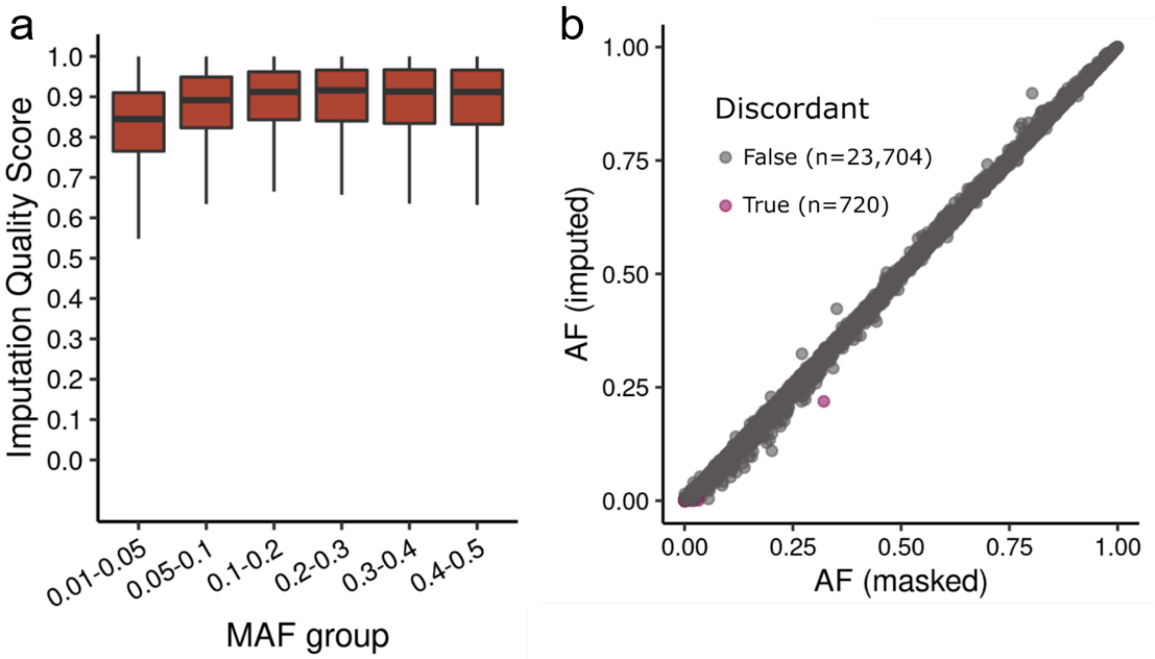

Supplementary Figure 1. Imputation quality. **a** Imputation Quality Score derived for masked variants for several minor allele frequency groups; **b** Allele frequency comparison between masked and imputed variants; MAF - minor allele frequency, AF - alternative allele frequency.

Supplementary Note 4.2 Validation of systematic sex-mismatch absence

Initially, we implemented a control procedure to ensure there were no systematic errors in our sample mapping. We created a pool of 44 pairs of relatives identified through the clinical records. We specifically looked for putative father-child pairs as they could be approximated by the matching last names and child's patronymic (first name of the father).
Out of 44 putative pairs, we successfully identified 31 first-degree relatives, 1 second-degree relatives. Remaining 12 pairs were followed by the clinical team of which 10 have confirmed the absence of the relatedness (random match between the names) and 2 pairs did not respond to the follow-up request. In total we confirmed 32 out of 32 suspected relatives and confirmed the absence of relatedness among 10 pairs that were not related. Which almost certainly excludes the possibility of systematic sample mismatch.

Next, we analyzed the pattern of sex mismatch observations in the 96-well plates that entered the genotyping pipeline. We identified 12 plates from a total of 43 that had more than 5 sex-mismatches each. We marked 937 samples on these 12 plates as potentially compromised due to the presence of sex-mismatched samples on the plates. We conducted a comparison of allele frequencies between these 937 samples and samples from the remaining 31 plates (**Sup. Fig. 2**). This analysis revealed that no systematic error was present among the samples.

Finally, we confirmed that new associations reported in our manuscript could not be attributed to the 96-well plate origin of the sample. We did so by comparing the allele frequencies of GWAS hits that were highlighted in our manuscript (**Sup. Fig. 24**). This analysis employed a logistic model that incorporated variables such as compromised or non-

compromised PLATE (0,1), number of alleles, phenotype, age, sex, and principal components (PC1 to PC4). Our analysis did not detect any statistically significant differences between allele frequencies for specific traits: Uric acid (rs4697701, p=0.296), LDL (rs7412, p=0.28), Abdominal obesity (rs56046524, p=0.537), Smoking initiation (rs13266066, p=0.65), Smoking status (rs7961991, p=0.389), and High blood pressure in the second half of pregnancy (rs11948871, p=0.054).

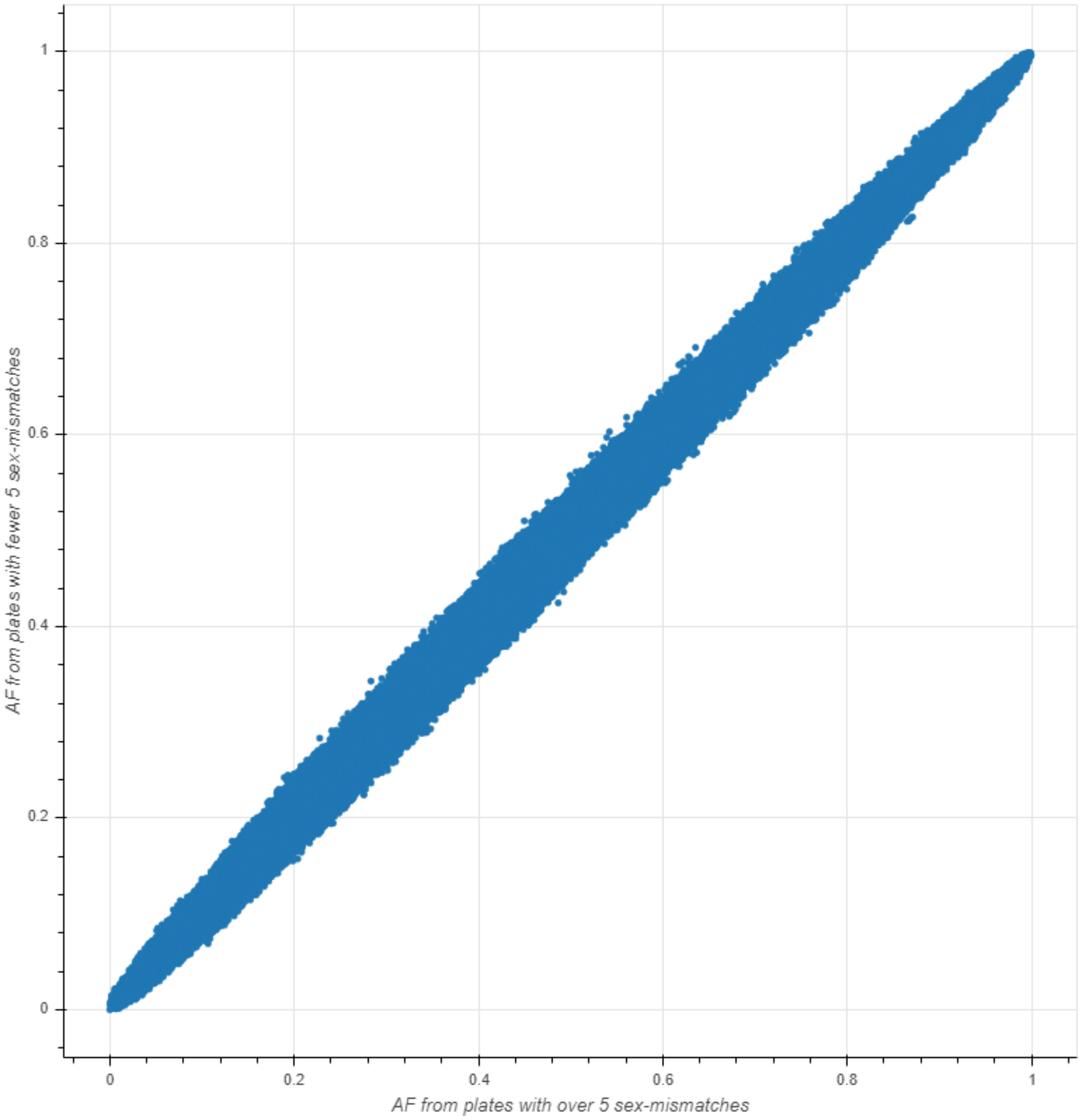

Supplementary Figure 2. Comparison between the allele frequencies of samples obtained from plates with over 5 sex-mismatches and those from plates with fewer than 5 sex-mismatches

Supplementary Note 4.3 Kinship analysis

We used PLINK2 [32] to identify relationships between individuals in the Russian population. We defined the 1st relationship degree as kinship value between 0.177 and 0.354 and second degree as value between 0.088 and 0.177. Thus, we had 400 pairs of the first degree relationship and 66 pairs of the second degree. We used *'igraph'* (v 1.2.11) R [33,34] package to visualize all relatives (**Sup. Fig. 3**). Then using PLINK2 kinship estimator we

marked 347 individuals among relatives in such a way that when they were excluded, only unrelated ties remained. Also, we identified 190 individuals with kinship more than 0.354 which correspond to twins. We defined these samples as duplicates and removed them from the study.

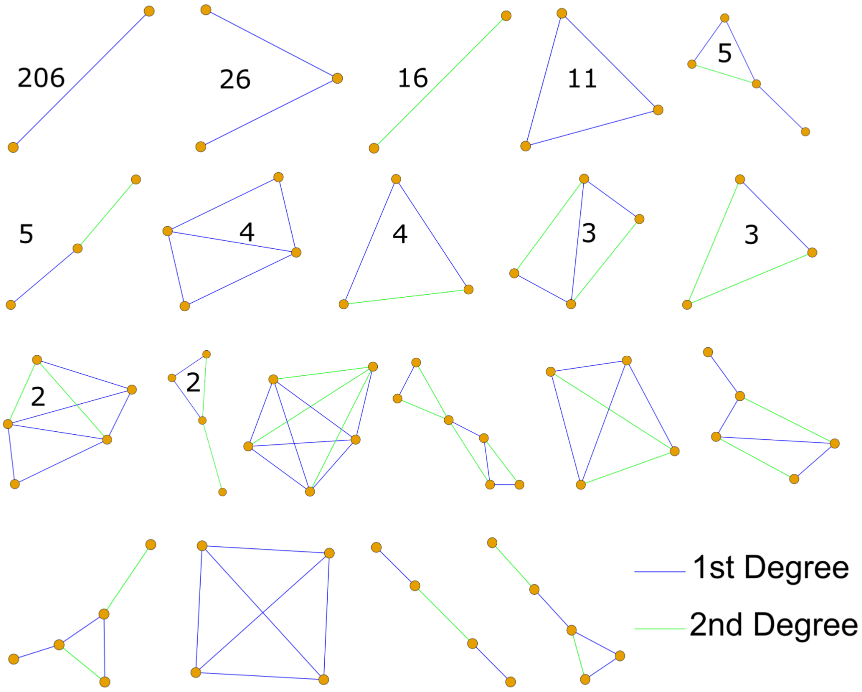

Supplementary Figure 3. All sets of relative groups in the combined ESSE-Starvation controls dataset. Only among ESSE cohort relatives were detected.

Supplementary Note 4.4 Genetic Data Quality Control

We used python3.6 *'hail'* (v0.2.85) [35] package to assess the variant quality control. Initially we removed 37,439 variants failing Hardy-Weinberg equilibrium ($p < 1x10^{-4}$) and 371 discordant variants with the HRC imputation panel. Discordant variants were defined as those with an allele frequency differing from the HRC panel by more than 0.15.

The distribution of MAFs and allele count for rare variants (MAF<0.01) is depicted in **Sup. Fig. 4a-b**. For directly genotyped variants, the majority of rare variants had an allele count of 0, whereas the full set of rare variants predominantly had allele counts ranging from 1 to 20 (**Sup. Fig. 4a-b**).

Next, we compare MAF of our Russian variants with MAFs from non-Finnish and Finnish gnomAD samples. We used gnomAD hail matrix and filtered all variants with call rate less than 0.97. Also, we filtered out all variants that did not pass gnomAD Random Forest filters. Therefore only 537,363 genotyped variants and 10,049,642 imputed variants were included into comparison. Comparison with non-Finnish Europeans for genotyped and all variants is shown at **Sup. Fig. 4c-d**, correspondingly. The comparison with Finns for genotyped and all variants is shown at **Sup. Fig. 4e-f**, correspondingly.

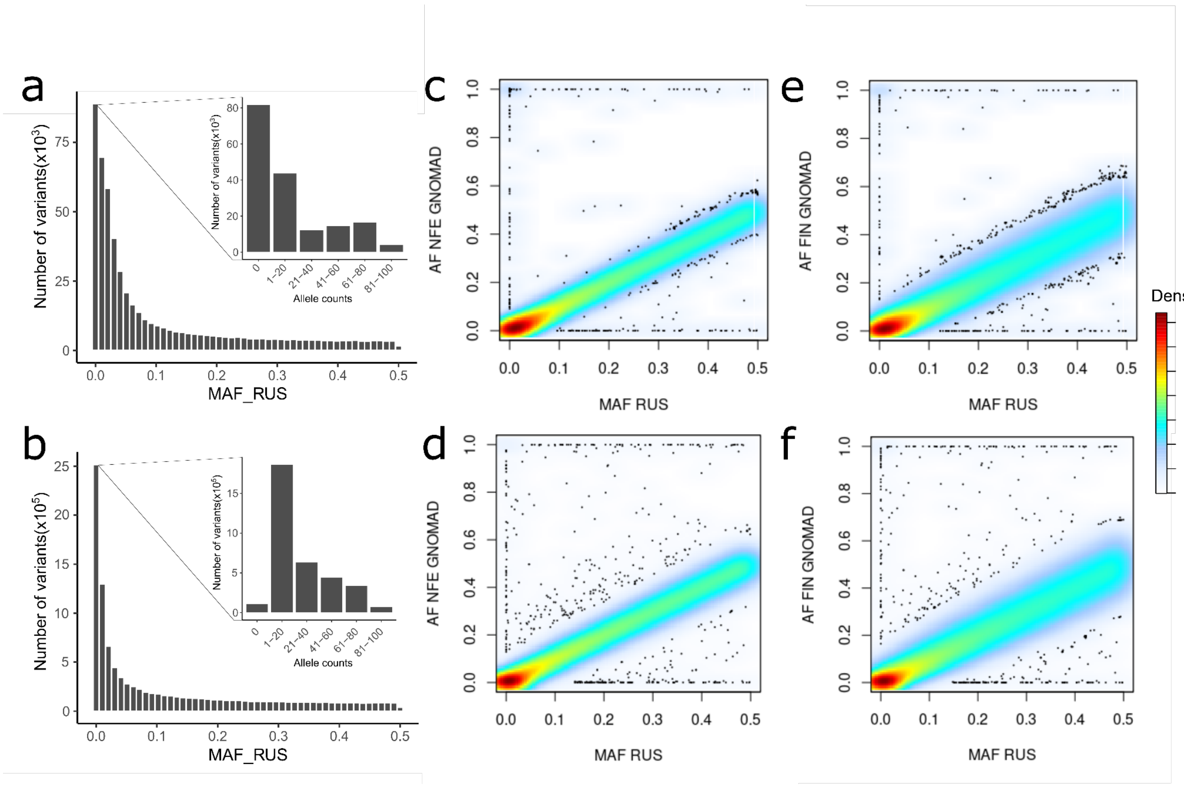

Supplementary Figure 4. Variant quality control. **a** Distribution of MAFs for genotyped variants and allele counts for rare variants (MAF<0.01); **b** Distribution of MAFs for all variants and allele counts for rare variants (MAF<0.01); **c** Comparison of genotyped MAFs in Russian population with allele frequencies in gnomAD Non Finnish Europeans; **d** Comparison of all MAFs in Russian population with allele frequencies in gnomAD Non Finnish Europeans; **e** Comparison of genotyped MAFs in Russian population with allele frequencies in gnomAD Finnish samples; **f** Comparison of all MAFs in Russian population with allele frequencies in gnomAD Finnish samples.

Supplementary Note 5 Principal Component Analysis

We used PCA analysis to stratify the Russian population using the PLINK2 tool. Initially we conducted pruning to find independent variants (R^2<0.2). 3,477,939 rare variants with MAF<0.01 were excluded. LD-pruning resulted in 535,727 independent variants (R^2<0.2). 4,281 individuals and 535,727 variants were used for PCA (**Sup. Fig. 5a-b**).

We used R library *'adamethods' (v1.2.1)* [36] to iteratively identify PC outliers in PC1-PC4. In each iteration we excluded 3 outliers with the highest distance to 5 nearest neighbors and recalculated LD-pruned variants and PCA. We stopped the procedures at step 45 when max Euclidean distance between two points was minimal (**Sup. Fig. 6**). Also, we excluded 1 individual from the African population according to further ADMIXTURE analysis. Therefore, the final dataset of PCA analysis included 4,145 individuals and 536,579 LD-pruned variants (**Sup. Fig. 5c-d**).

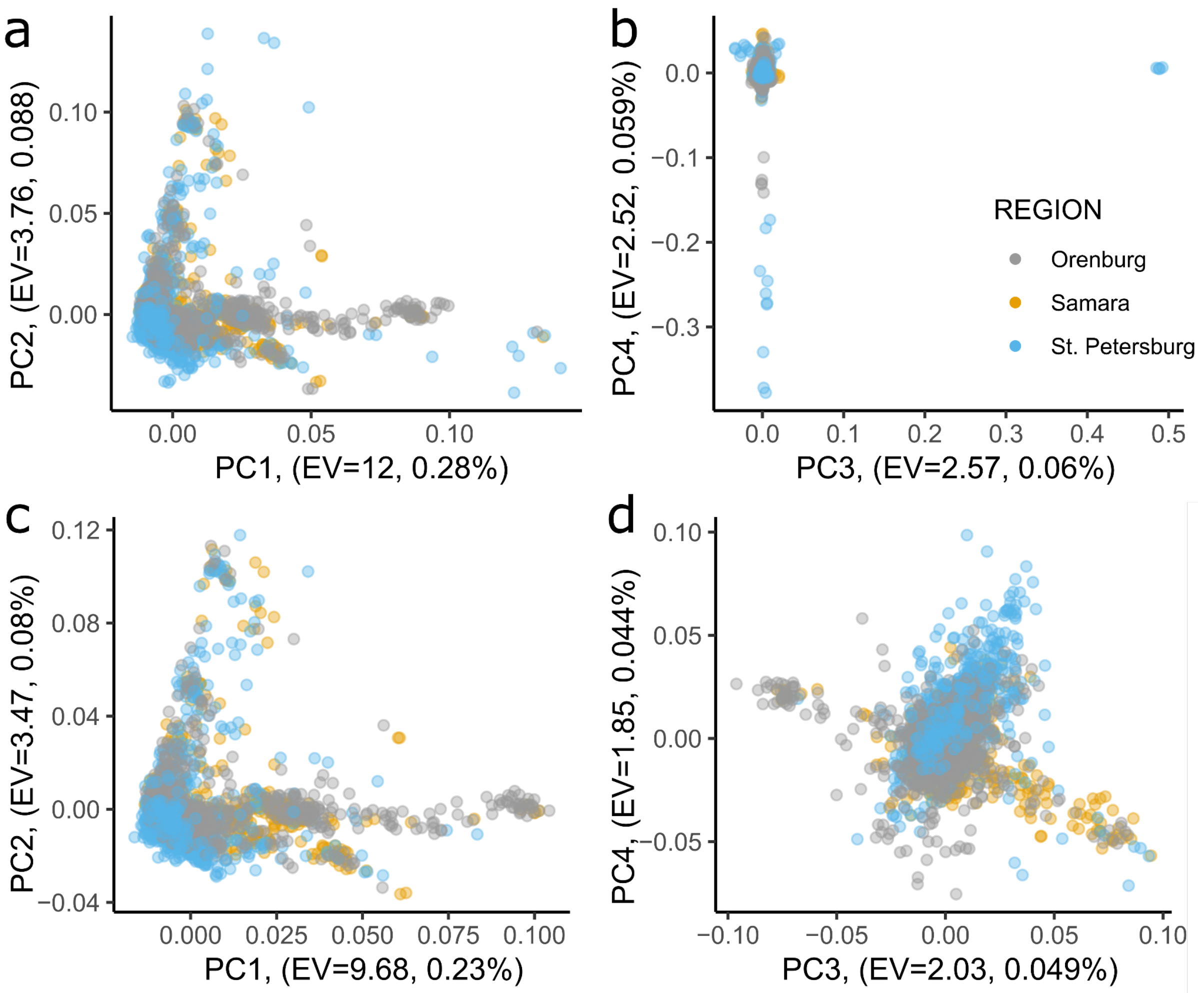

Supplementary Figure 5. Principal component analysis. **a** PC1 and PC2 for Russian population before filtering outliers; **b** PC3 and PC4 for Russian population before filtering outliers; **c** PC1 and PC2 for Russian population after filtering outliers; **d** PC3 and PC4 for Russian population after filtering outliers

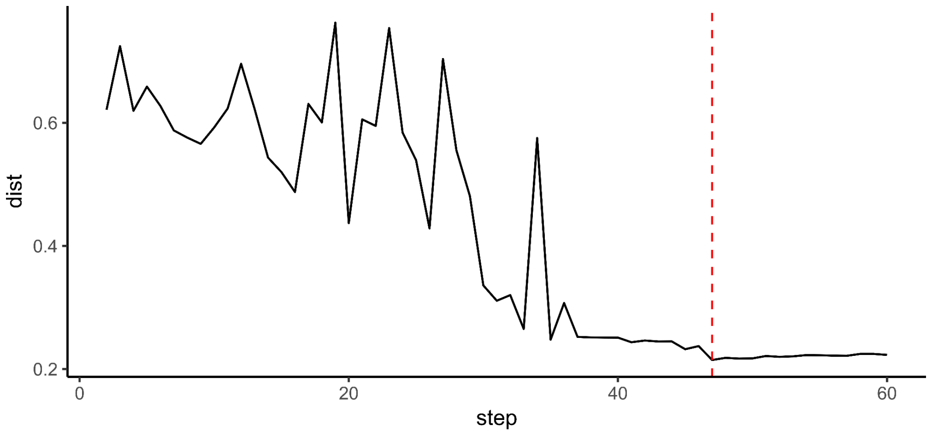

Supplementary figure 6. Maximum Euclidean distance in each iteration of filtering outliers

Supplementary Note 6 Comparison of Russian samples with 1000 Genomes

Using python3.6 *'hail'* (v0.2.85) package we combined the Russian initial dataset (4,281 individuals and 11,077,392 variants) with 1000 Genomes WGS data which contained 10,267,739 variants and 2,504 individuals. We filtered variants with MAF <0.01 and HWE > $1x10^{-4}$ and conducted pruning (R^2<0.2). This resulted in 506,617 LD-pruned variants and 6,785 individuals that were used to build PCs (**Sup. Fig. 7a**). The PCA without 136 Russian outliers are shown at **Fig. 2b**. The similar procedure was carried out with 1000 Genomes Europeans. Combined genotyping data contained 10,267,739 variants and 4,784 individuals. 515,649 LD-pruned variants were used to build PCs. We merged Utah residents (CEU) with Northern and Western European ancestry with British Europeans as they are genetically close. Also, we combined Spain and Italy populations (**Sup. Fig. 7b**). The PCA without 136 Russian outliers is shown at **Fig. 2c** and **Sup. Fig. 8**.

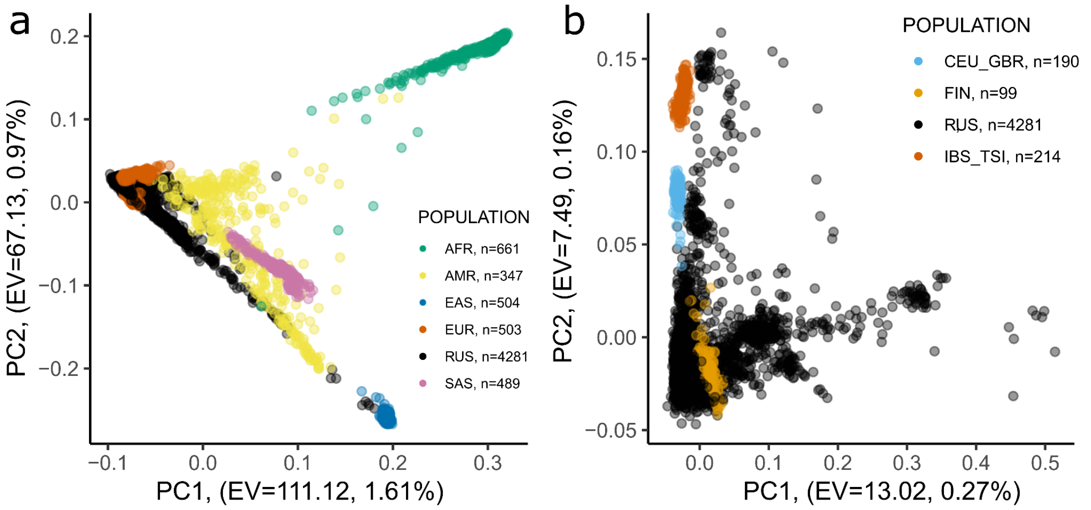

Supplementary Figure 7. Principal component analysis of Russian samples combined with 1000 Genomes without filtering Russian outliers. **a** PC1 and PC2 for combined Russian and 1000 Genomes dataset; **b** PC1 and PC2 of Russian samples combined only with 1000 Genomes Europeans.

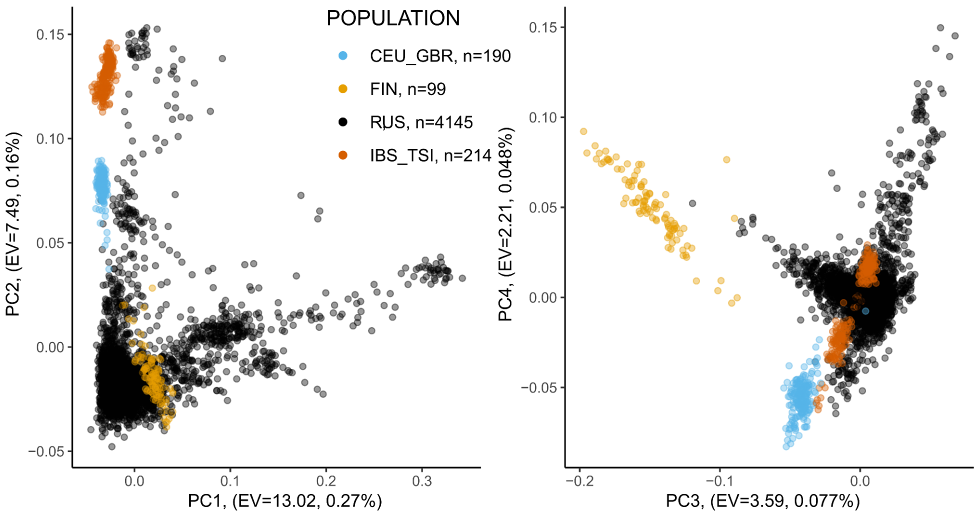

Supplementary Figure 8. Principal component analysis of Russian samples combined with 1000 Genomes Europeans: PC1:PC4.

Supplementary Note 7 Clustering of Russian samples

We used the '*SVDFunctions' (v1.2)* [37] R package to identify clusters in the Russian population. We selected the number of clusters in such a way that they contained more than 100 individuals and were interpretable in the first 2 principal component spaces **(Fig. 2d)**. The resulting dendrogram with 6 clusters is shown at **Sup. Fig. 9a** and cluster enrichment among geographical regions are shown at **Sup. Fig. 9b**.

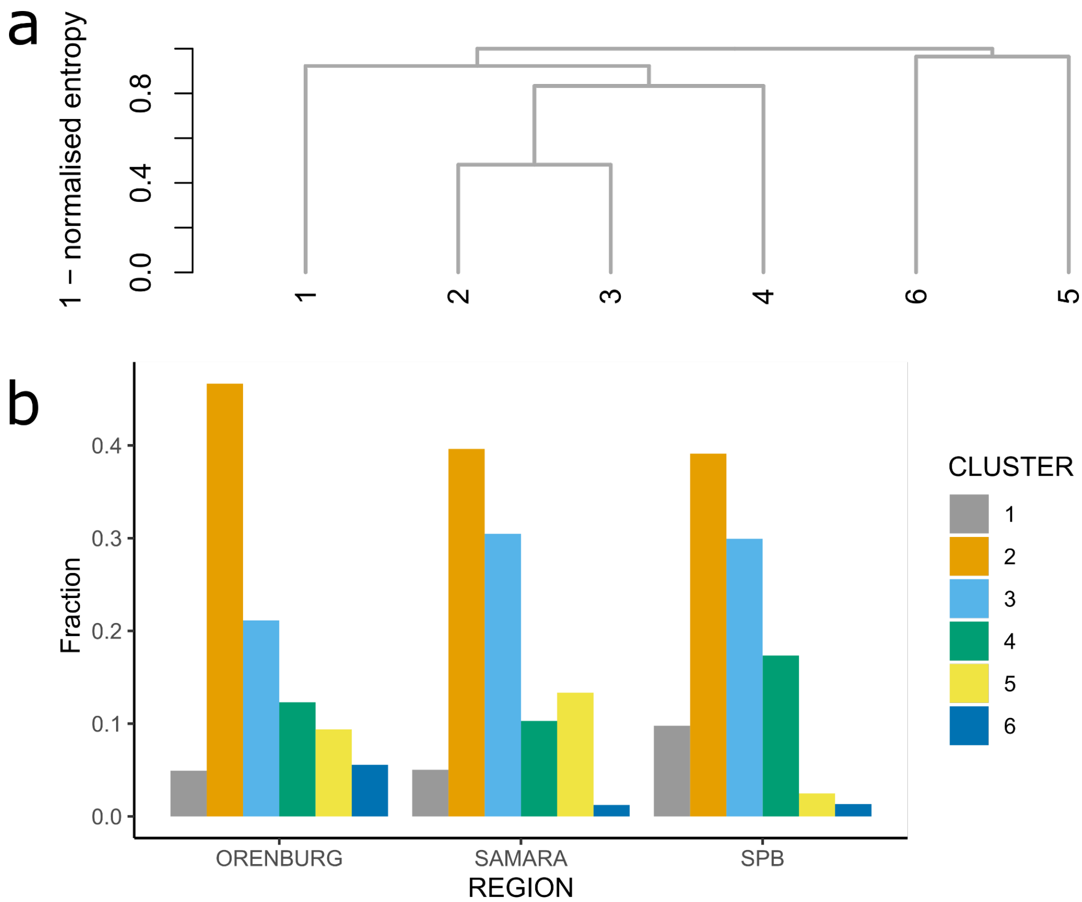

Supplementary Figure 9. Clustering of Russian samples based on PC1-PC4. **a** Hierarchy of clusters; **b** Enrichment of clusters in each geographical region.

Supplementary Note 8 Admixture analysis

To assess the genetic makeup of the Russian population we run ADMIXTURE (v1.3.0) [38] in supervised mode on a LD-pruned combined (ESSE/Starvation Controls - 1000 Genomes) dataset with 506,617 LD-pruned variants and 6,649 samples. Initially we identified an additional 14 relatives in the 1000 Genomes dataset using the PLINK2 tool and excluded all 361 related individuals from analysis. Training dataset included 8 populations form 1000 Genomes: African (AFR, N=652), American (AMR, N=347), Vietnamese (CDX_KHV, N=192), Utah residents (CEU) with Northern and Western European ancestry and British (CEU_GBR,

N=190), Chinese and Japanese (CHB_CHS_JPT, N=312), Finns (FIN, N=99), South Europeans (IBS_TSI, N=214) and South Asians (SAS, N=484). For each individual in the Russian dataset, we determined the percentage of each training population and arranged them by PC1 (**Fig. 2e**). The distribution of each training population for each cluster is shown at **Sup. Fig. 10.** Admixture analysis using only HapMap variants is provided in **Sup. Fig. 11.**

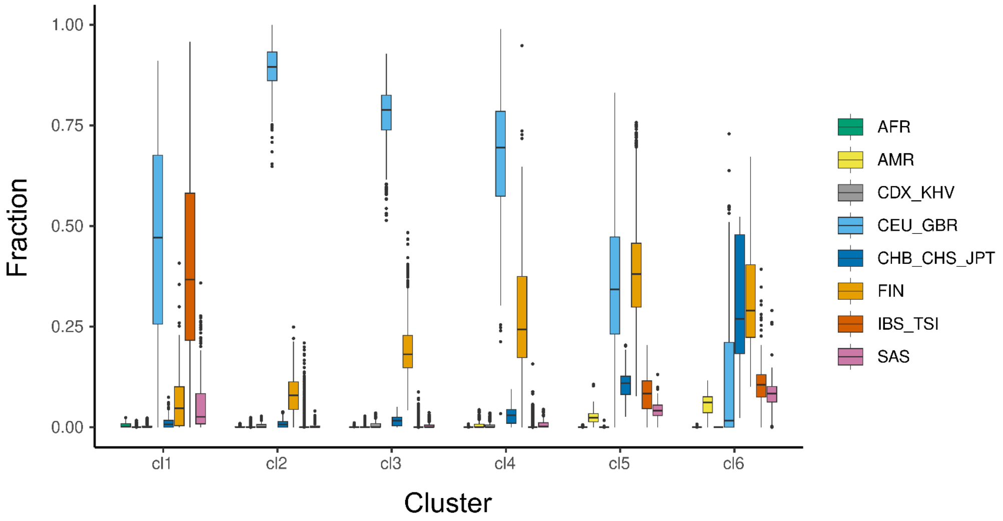

Supplementary Figure 10. Distribution of each analyzed population by clusters. Each boxplot represents the minimum, first quartile (Q1), median (Q2), third quartile (Q3), and maximum values.

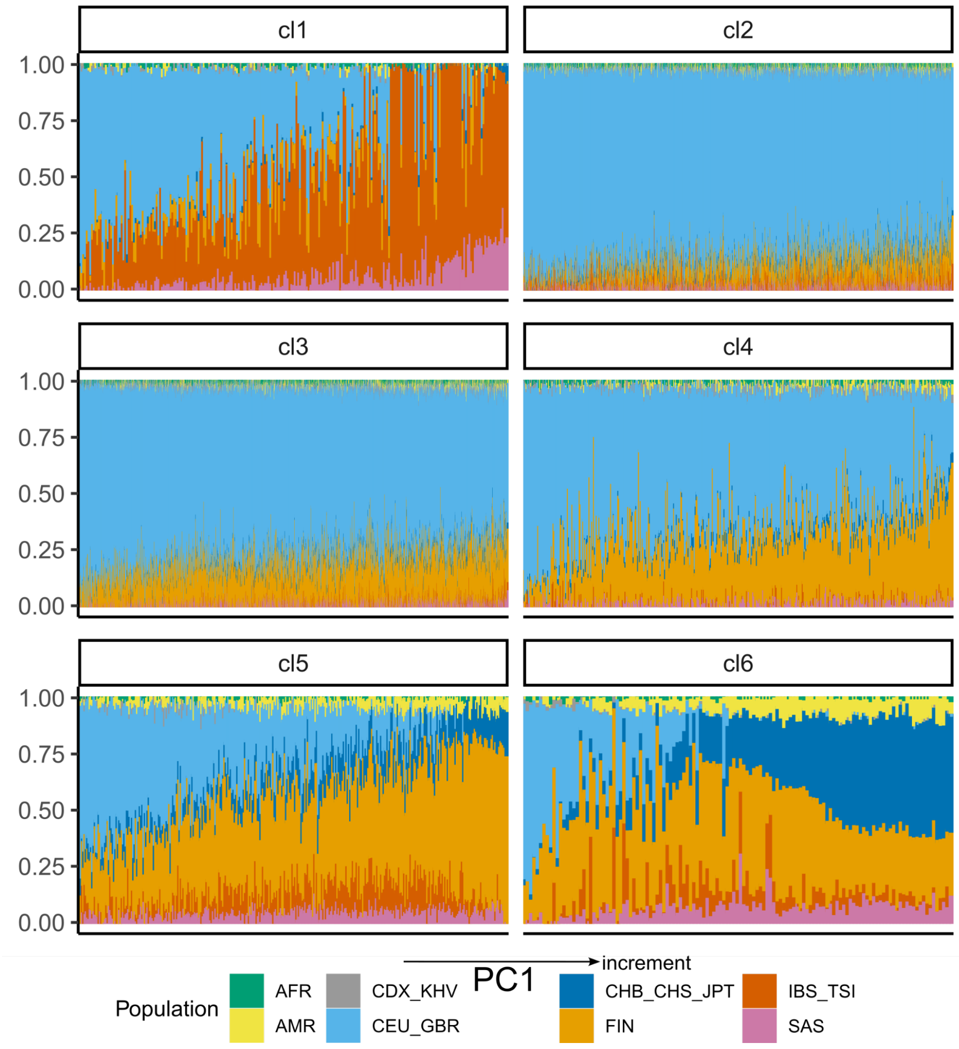

Supplementary Figure 11. Admixture analysis using only HapMap variants. Each bar represents one individual

Supplementary Note 9 F_{st} estimation

To estimate the relationship between the Russian population and other populations from 1000 Genomes we also calculated the Fst metric. Initially, we used VCFtools (v0.1.15) [39] to calculate F_{st} between Russian geographical regions and 1000 Genomes populations using a LD-pruned genotype matrix (506,617 variants and 6,649 individuals). We excluded 361 related individuals and calculated mean F_{st} for each chromosome independently. The distribution of resulting Fst values is shown at **Sup. Fig. 12**. Also, we calculated the Fst between each Russian cluster and each 1000 Genomes population. Corresponding distributions are shown at **Sup. Fig. 13**.

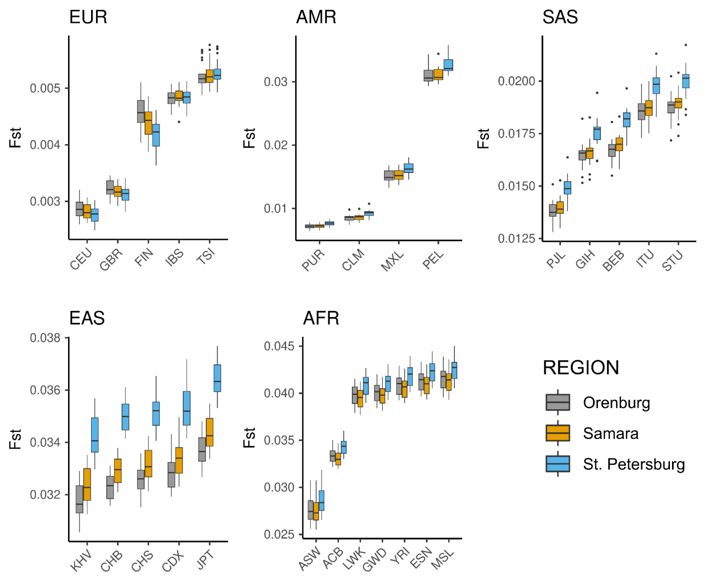

Supplementary Figure 12. Fst estimation between each geographical region in the Russian dataset and 1000 Genomes populations. Each boxplot represents the minimum, first quartile (Q1), median (Q2), third quartile (Q3), and maximum values.

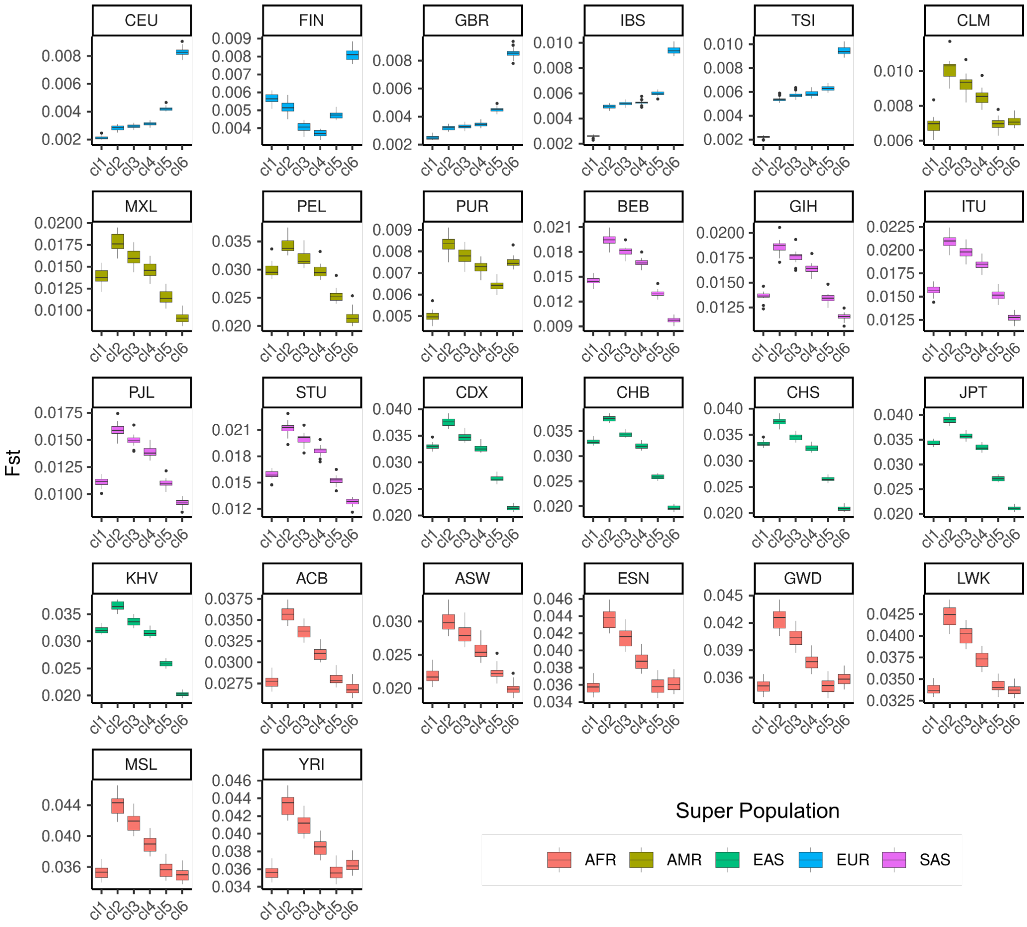

Supplementary Figure 13. Fst estimation between each Russian population cluster and 1000 Genomes populations. Each boxplot represents the minimum, first quartile (Q1), median (Q2), third quartile (Q3), and maximum values.

Supplementary Note 10 Identity-by-descent (IBD) estimation

We utilized BEAGLE 4.0 (beagle.r1399.jar) [40] to compute IBD-sharing statistics between each pair of individuals in the combined (ESSE/Starvation Controls - 1000 Genomes) LD-pruned dataset. We collected only IBD regions with ibdlod quality score more than 3. Then we used 'merge-ibd-segments.17Jan20.102.jar' to merge IBD segments if the gap between segments had at most one discordant homozygote and that was less than 0.6 cM in length. Individuals identified as related (N=361) were excluded from the analysis. The total length of all IBD-segments (in cM) was calculated for each pair of individuals, and then the median length was computed across each pair of populations. We calculated Euclidean distances to make clasterization and used '*gplots'* (v3.0.3) [41] to visualize heatmap matrix (**Fig. 2f**)**.** IBD-searing statistics between each 1000 Genomes population and Russian clusters is shown at **Sup. Fig. 14.** Maps were created using the basemap toolkit from Python3.6 '*matplotlib*' (v3.5.2) library [42]

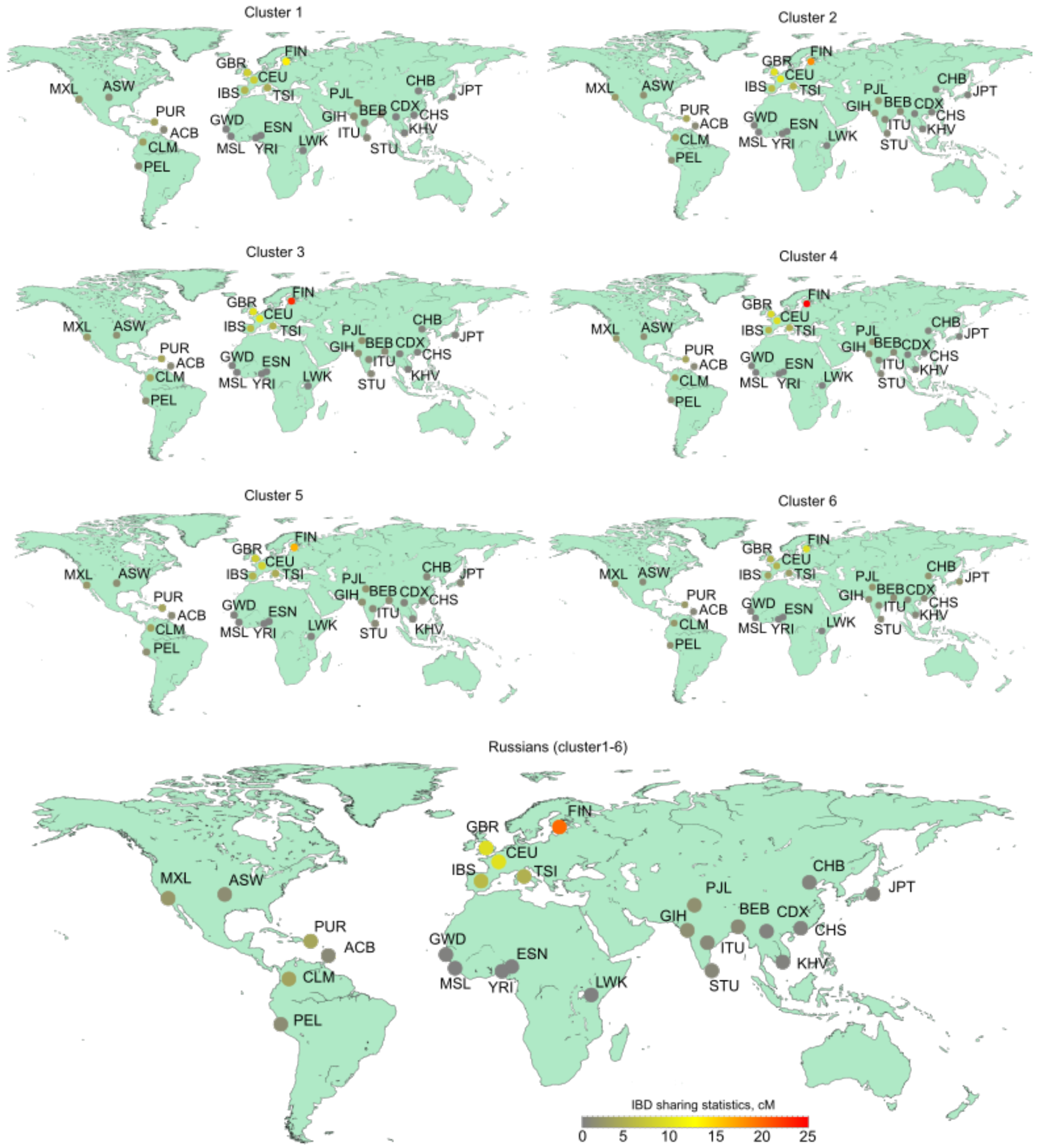

Supplementary Figure 14. Mean IBD length between Russian population (cl1-cl6) and 1000 Genomes populations. The map was generated with the Matplotlib Basemap toolkit for Python (https://pypi.python.org/pypi/basemap; © 2011-2014 Jeffrey Whitaker © 2015-2024 The Matplotlib development team; Basemap toolkit for Python (https://pypi.python.org/pypi/basemap). Coordinates of 1000 Genomes populations were download from IGSR portal (https://www.internationalgenome.org/data-portal/population).

Supplementary Note 11 Population analysis utilizing only genotyped variants

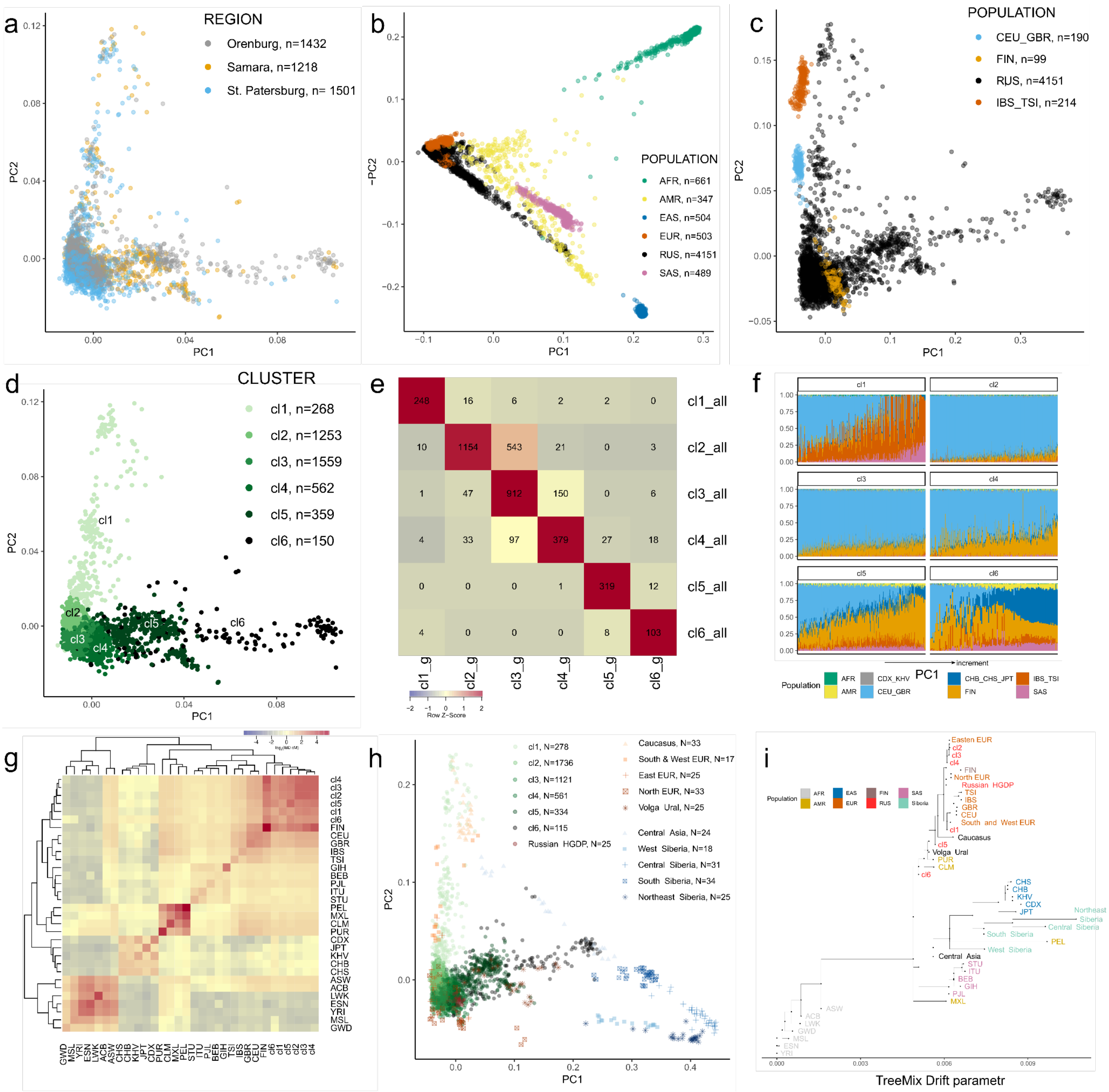

Supplementary Figure 15. Populational analysis utilizing only genotyped variants. **a** Principal component analysis of the Russian population with labeling indicating sample collection region; **b** Joint principal component analysis of the Russian population with 1000 Genomes cohort; **c** Joint principal component analysis of the Russian population with European subsample of 1000 Genomes cohort; **d** Clustering of the Russian cohort in the PCA space; **e** Correlation matrix between classters identifying using all variants and only high quality genotyped variants; **f** Admixture analysis for each of six populational clusters in the Russian cohort, samples were arranged with respect to their PC1 coordinate; **g** Hierarchical clustering of the Russian cohort with 1000 Genomes subpopulations with respect to the sharing of IBD regions; **h** Joint principal component analysis with Russian population and several neighboring populations from EGDP; **i** TreeMix analysis of the relatedness between 1000 Genomes subpopulations, EGDP populations, HGDP Russians and clusters within the Russian cohort.

Supplementary Note 12 Enrichment of Finnish and Russian variants

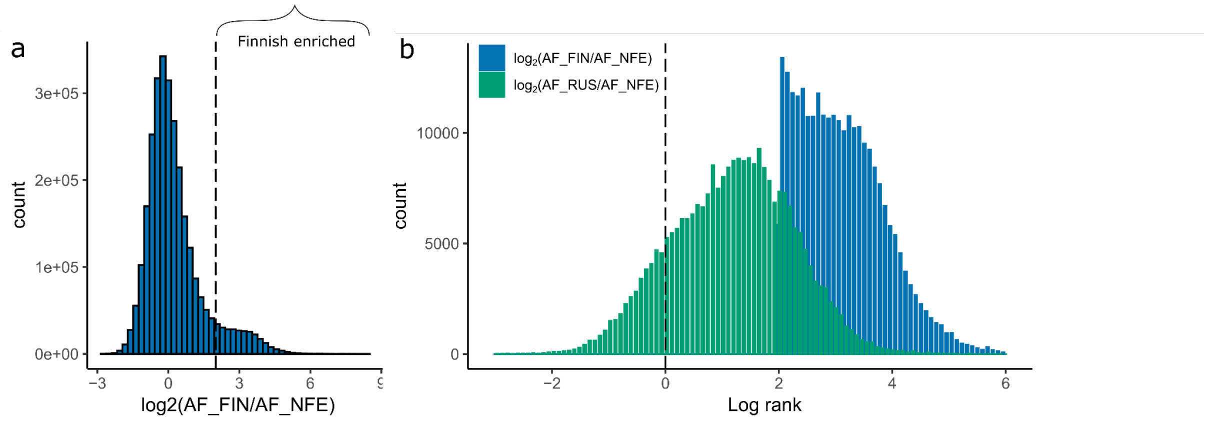

Supplementary Figure 16. Finnish enriched variants. **a** Logarithmic ratio of frequencies in Finnish to non-Finnish Europeans for all variants with Finnish AF (0.01-0.1); **b** Comparison of frequencies of Finnish enriched variants in the Russian population.

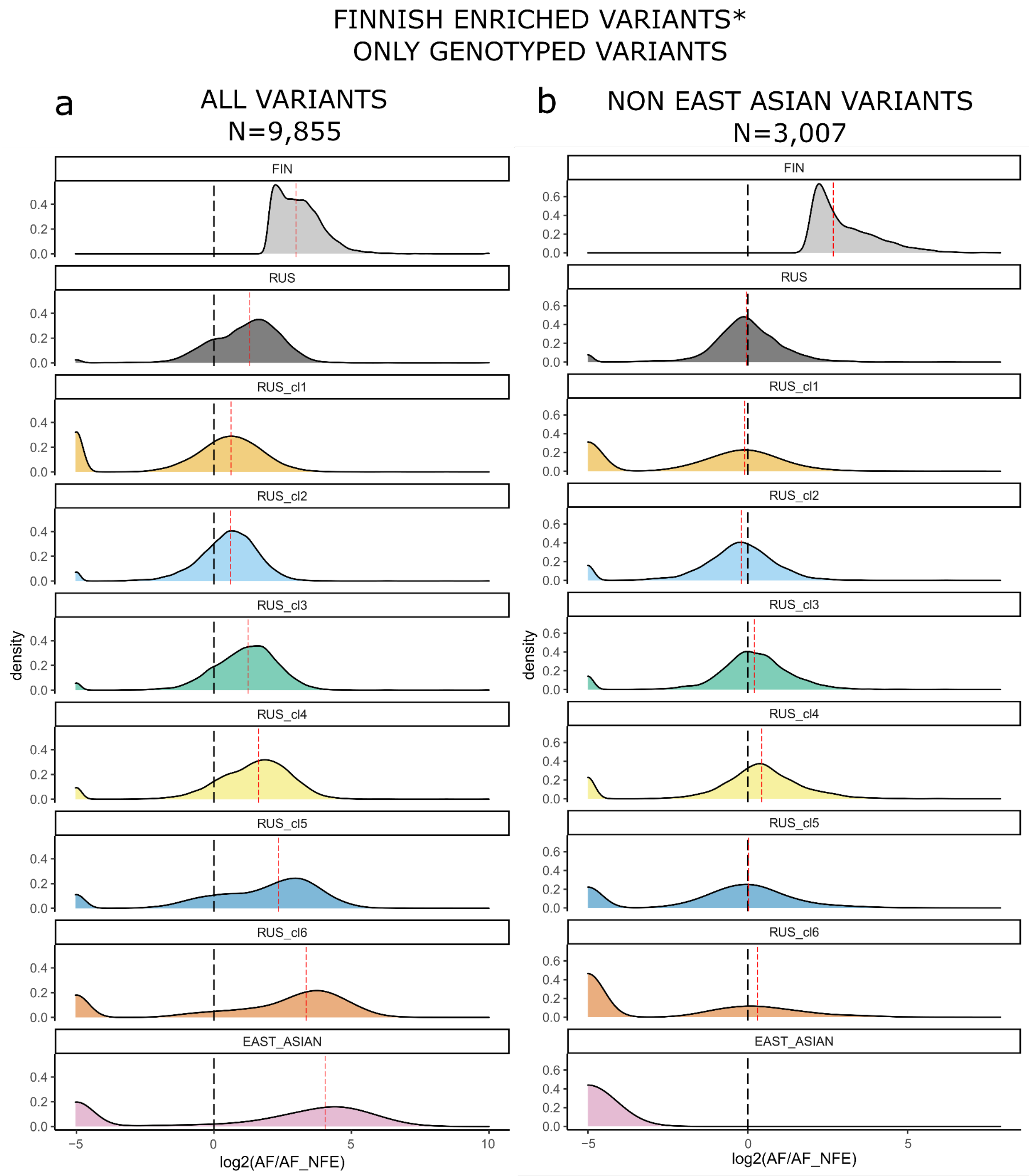

Supplementary Figure 17. Finnish enriched variants in the Russian pupolation. **a** The distribution of enrichment of genotyped Finnish-enriched variants across clusters in the Russian cohort, Finnish and East Asian cohorts from gnomAD; **b** Distribution of enrichment of the Finnish-enriched variants that are not found in East Asian population in gnomAD across clusters in the Russian and Finnish cohorts from gnomAD. Red dashed line shows the median of the distribution.

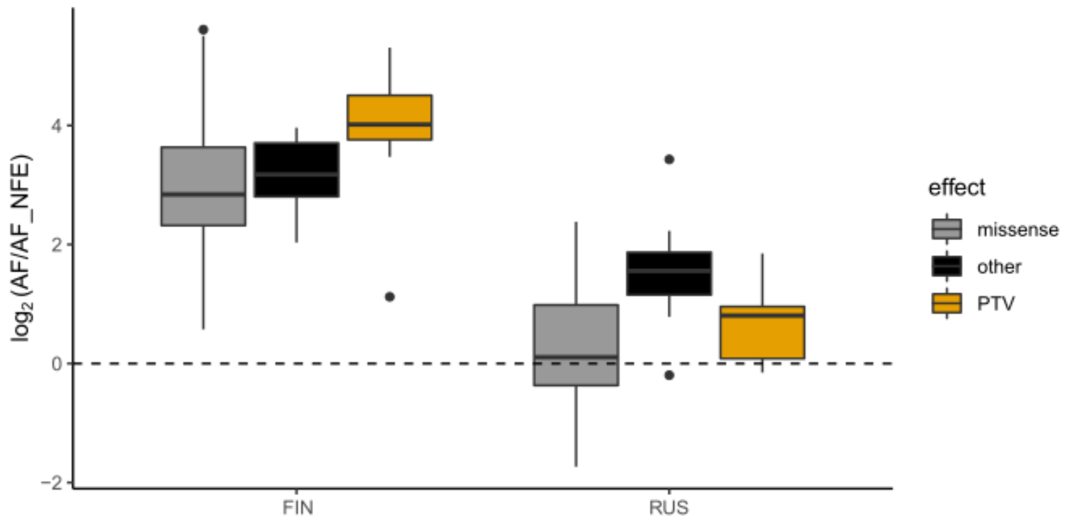

Supplementary Figure 18. Previously reported Finnish enriched variants that were associated with clinical phenotypes [28]. Each boxplot represents the minimum, first quartile (Q1), median (Q2), third quartile (Q3), and maximum values.

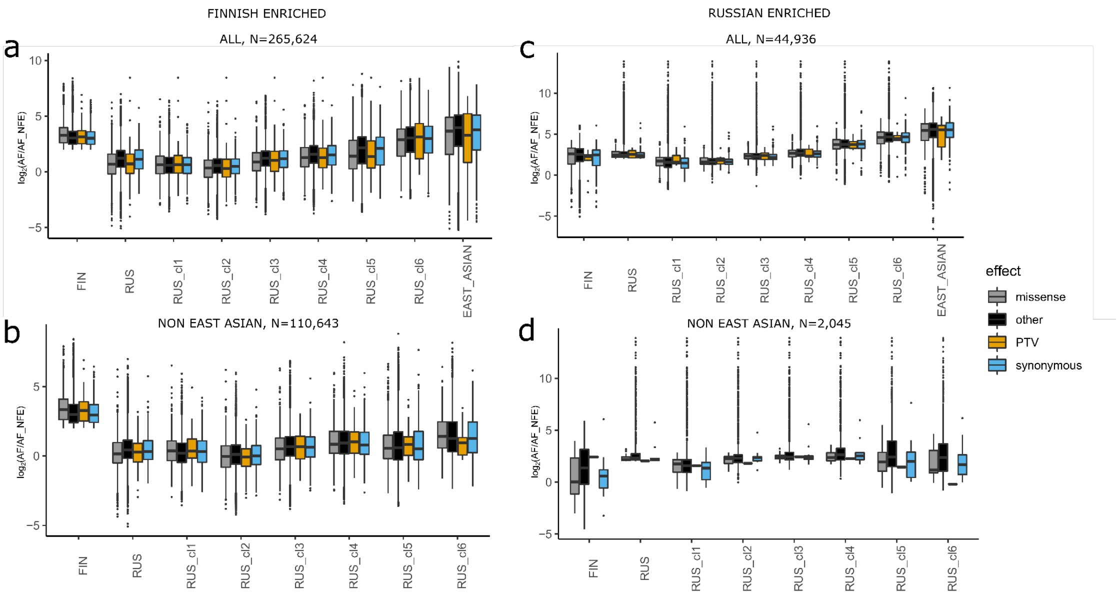

Supplementary Figure 19. Finnish and Russian enriched variants. **a** All Finnish enriched variants; **b** Non East Asian Finnish enriched variants; **c** All Russian enriched variants; **d** Non East Asian Russian enriched variants. Each boxplot represents the minimum, first quartile (Q1), median (Q2), third quartile (Q3), and maximum values.

Supplementary Note 13 Estimated population size

Estimated population size was calculated for resulting IBD regions with length more than 2cM using IBDne (ibdne23Apr20.ae9.jar) tool[43] for Finnish and Russian populations (**Fig. 3c**). Estimated population size for pairs of clusters in comparison with Finnish populations are shown at **Sup. Fig. 20.**

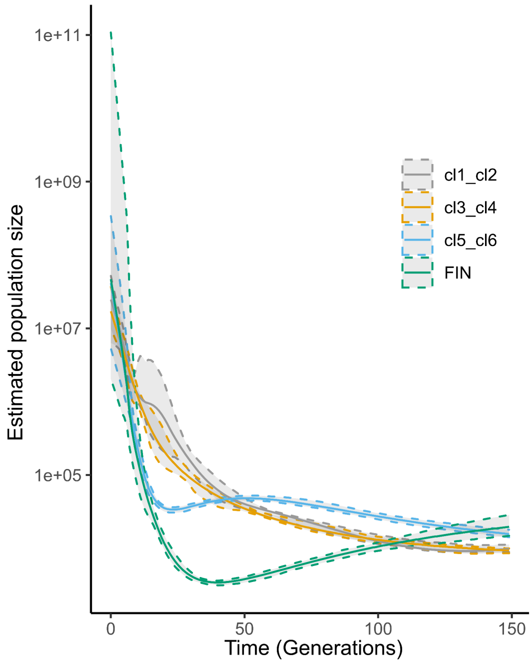

Supplementary Figure 20. Estimated population size, error bands show 95% bootstrap confidence interval.

Supplementary Note 14 Population Tree Construction

6,288 unrelated Russians and 1000 Genomes individuals were used to construct the maximum likelihood tree based on the combined (ESSE/Starvation Controls - 1000 Genomes) LD-pruned dataset. Additionally we included several populations from the Estonian Genome Diversity Panel (EGDP): **Caucasians** (*Abkhazians, Avars, Azerbaijanis, Balkars, Circassians, Georgians, Kabardins, Kumyks, Lezgins, North-Ossetians, Tabasarans*, N=33), **Europeans** (**East** (*Poles, Belarusians, Ukrainians_east, Ukrainians_north, Ukrainians_west, Cossacks, Cossacks_Kuban, Russians-Central, Russians-North, Russians-West,Russians*): N=25; **North** (*Swedes, Estonians, Finnish, Latvians, Lithuanians, Saami, Ingrians, Karelians, Vepsas, Mordvins*): N=33; **South & West** (*Germans, Hungarians, Moldavians, Albanians, Roma, Croats*): N=17; **Volga Ural** (*Maris, Udmurds, Komis, Tatars, Mishar-Tatars, Kryashen-Tatars, Bashkirs, Chuvashes*): N=25), **Siberians** (**West** (*Mansis, Khantys, Forest-Nenets, Tundra-Nenets, Selkups, Kets*): N=18; **Central** (*Nganasans, Sakha, Evens_Sakha, Evens_Magadan, Evenks, Yakuts*): N=31; **South** (*Shor, Altaians, Tuvinians, Buryats, Mongolians*): N=34, **Northeast** (*Koryaks, Chukchis, Eskimo*), N=25) and **Central Asians** (Rushan-Vanch, Shugnan, Tajiks, Yaghnobi, Turkmens, Uzbek, Kyrgyz_Tdj, Kyrgyz, Kazakhs, Uygurs, Ishkasim): N=24) [44]. Also we added a Russian population (N=25) from the Human Genome Diversity Panel [45]. PCA for Russians, HGDP Russians and several

populations from EGDP is shown at **Sup. Fig. 21**. Populational tree based on allele counts for each population was constructed using TreeMix (v.1.12), with the genome split into blocks of 500 SNPs and using default bootstrapping (**Fig. 3d**) [46]. A population tree that includes HGDP populations is shown at **Sup. Fig. 22**

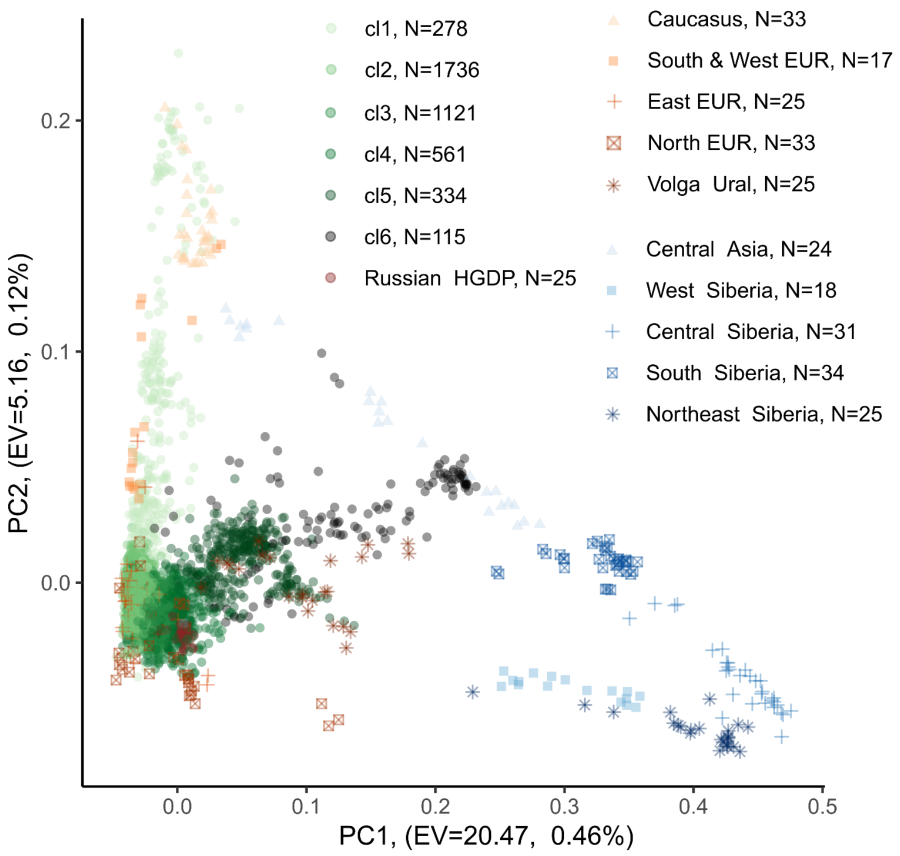

Supplementary Figure 21. PCA with the Russian population and several neighboring populations from EGDP.

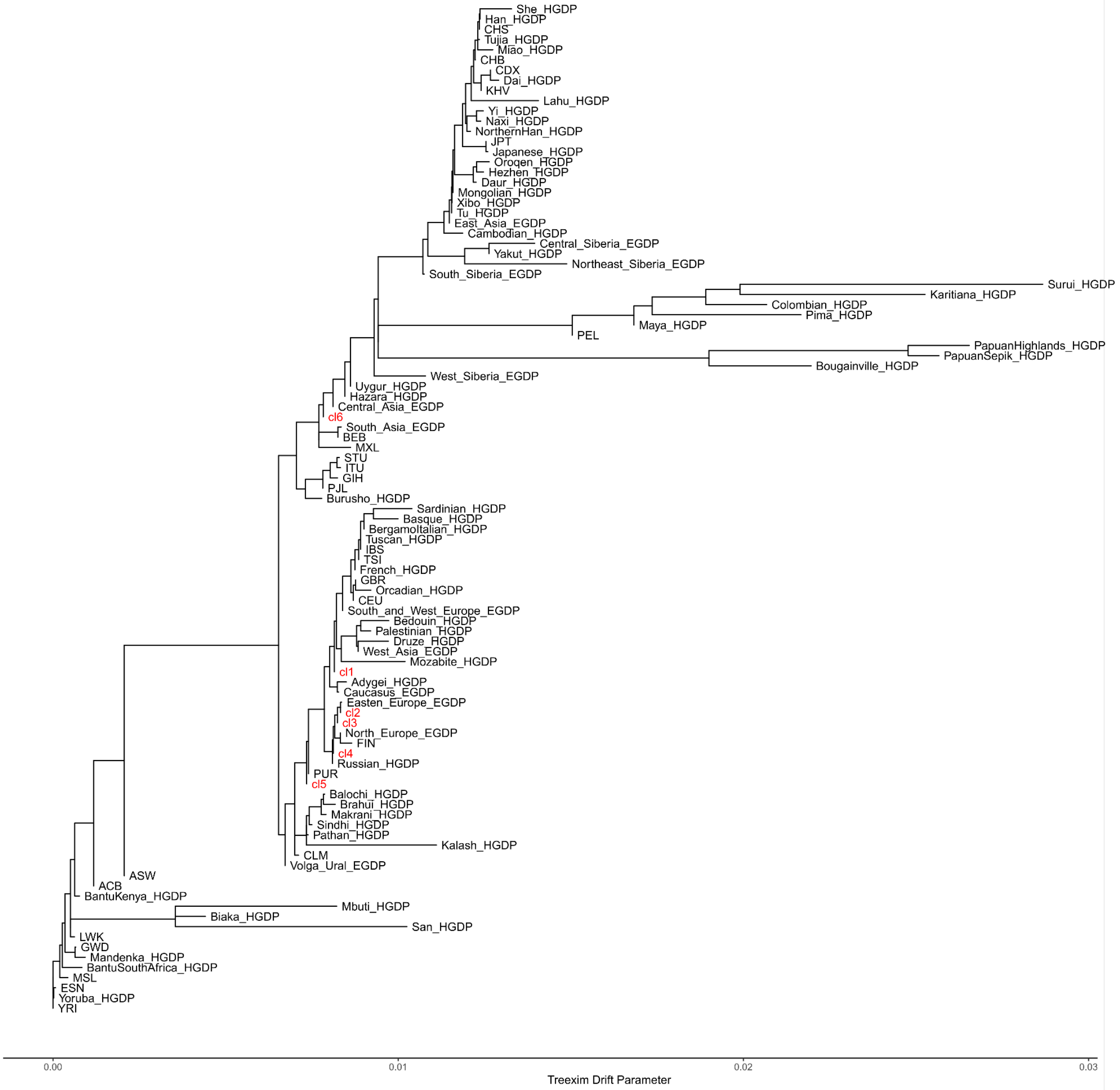

Supplementary Figure 22. Treemix analysis with additional populations from HGDP.

Finally we run ADMIXTURE analysis in an unsupervised mode with 8 genetic clusters and all (N=7,744) unrelated individuals from the Russian dataset, 1000 Genomes, HGDP and EGDP. Only local EGDP ethnicities and 1000 Genome populations are shown at **Sup. Fig. 23.**

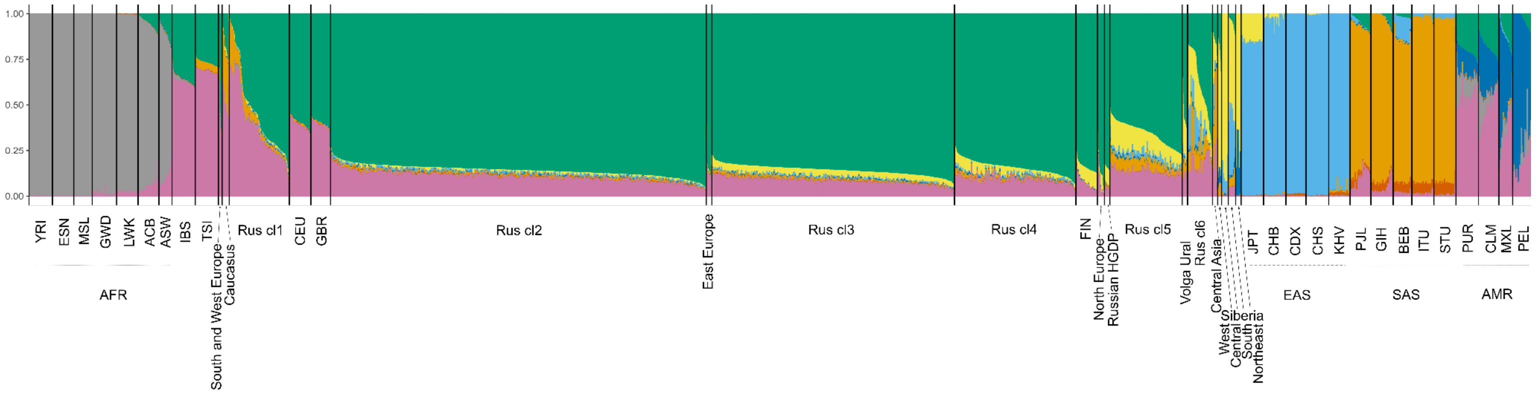

Supplementary Figure 23. ADMIXTURE analysis in unsupervised mode with 8 clusters.

Supplementary Note 15 GWAS

We performed GWAS for 464 phenotypes. We used linear models for continuous and categorical phenotypes and logistic models for binary phenotypes. Each GWAS model was adjusted for age, sex and PC1-PC4 and created using python3.6 *'hail'* (v0.2.85) package. Since some phenotypes were not determined for all individuals, additional quality control (MAF>0.01, HWE>0.0001) was performed separately for each GWAS. First, we checked well-known associations for LDL-cholesterol (N=3,816) and blood uric acid levels (N=3,757) to ensure the quality of the phenotyping. We found a strong association of the rs7412 variant (gene *APOE,* beta = -0.43, p=4.7×10^{-29}) and rs4970834 (gene *CELSR2,* beta= -0.15 ,p=1.81×10^{-8}) with LDL levels. rs7412 and rs4970834 were known variants associated with LDL levels in UK Biobank (beta = -0.51, p=0) and (beta=-0.14,p=0), correspondingly (**Sup. Fig. 24a**). For blood uric acid we found leading variants rs4697701 (gene *SLC2A9,* beta = -22.2, p=2.21×10^{-28}) and rs45499402 (gene *ABCG2,* beta = 21.9, p=3.99×10^{-12}) that were replicated in UK Biobank (beta = -22.7, p=0) and (beta=14.1,p=0), correspondingly (**Sup. Fig. 24b**).

Moreover, we confirmed an association of rs13266066 (AF RUS=0.4358, AF NFE = 0.4763, AF FIN = 0.4397) with smoking initiation that was previously predicted by MTAG algorithm (beta = -0.007, p=1×10^{-10}, beta was reversed to match models), while initial association was not strong (beta = -0.012, p=0.0037, beta was reversed to match models) [47]. In our data we found that the allele frequency of rs13266066 was significantly lower in a group of individuals who never smoked (N never smoked = 2,391; AF never smoked = 0.414, N controls = 1,488; AF controls = 0.475, beta = -0.28, p=3.74×10^{-8}) (**Sup. Fig. 24c**). In the UK biobank rs13266066 was nominally negatively associated with past tobacco smoking, never smoking and age of smoking initiation (p=1.5×10^{-7}, p=2×10^{-4},p=3×10^{-4}), correspondingly.

To reduce the possibility of technical artifacts associated with this observation we looked at only directly genotyped variants in this locus and confirmed the presence of highly-associated rs11781072 (p=1.45×10^{-6}). eQTL properties of rs13266066 increased expression of *PTK2* gene in a cerebrum (p=8.8×10^{-12}).

We made gene prioritization with GPrior [48] for *PTK2* using 251 genes associated with trait 'Smoking initiation (ever regular vs never regular)' (p<6×10^{-6}) from GWAS catalog as training set and GWAS summary statistic 'Smoking never' from UK Biobank (phenocode: 20116_0). We used only variants with p value less than 1×10^{-3} that were initially independently

(--rsID option) annotated by genes with POSTGAP and then prioritized with GPrior (**Sup. Fig. 25**). The results of prioritization showed that the *PTK2* was most likely associated with never smoking status according to all prediction models except SVM.

We also found an association rs7972723 (AF RUS=0.1465, AF NFE = 0.1447, AF FIN = 0.1680) with the current smoking status (N current smoker = 834, AF current smoker = 0.189, N controls = 3,045; AF controls = 0,137, beta = 0.43, p=2.08x10^{-8}) (**Sup. Fig. 24d**). Current smokers included both people who are currently smoking and who quit smoking less than 1 year ago. Interestingly, allele frequency of rs7972723 increased in three groups of smoking status: never smoked - 0.136, smoking in the past - 0.141, current smoker - 0.189 (p=1.63x10^{-7}). eQTL properties of rs7972723 increase expression of *ACSM4* gene in adipose subcutaneous tissue (p=1.7x10^{-7}). rs7972723 was nominally replicated in UK Biobank as associated with smoking in the past (beta=0.0036, p=0.02). Significantly associated directly genotyped variant rs7953422 also presented in this locus (p=3.54x10^{-8}). We didn't perform gene prioritization for *ACSM4* due to a small training gene set for smoking cessation (N=21).

In addition, we found some new associations. For example variant rs56046524 (AF RUS=0.3537, AF NFE = 0.3938, AF FIN = 0.3632) associated with abdominal obesity (N cases=1,405; AF cases = 0.306, N controls = 2,462; AF controls=0.378, beta=-0.324, p=3.7x10^{-9}) (**Sup. Fig. 24e**). Also variant rs11948871 (AF RUS=0.2024, AF NFE =0.1727, AF FIN = 0.1479) was associated with increased blood pressure in the second half of pregnancy (N cases=366; AF cases=0.279, N controls = 1,642; AF controls = 0.185, beta=0.55,p=1.4x10^{-8}) (**Sup. Fig. 24f**). Both these observations were accompanied with highly associated directly genotyped variants (rs100016, p=4.69x10^{-7}; rs6872733, p=2.21x10^{-5}), correspondingly.

We conducted a study to explore the genetic correlations between phenotypes from the Russian Biobank and their corresponding counterparts in the UK Biobank and FinnGen datasets. Initially, we excluded all GWAS with unreliable heritability estimates (confidence interval outside the range of [0,1]) and a heritability standard error exceeding 50%. This led to a total of 35 GWAS, 26 of which were matched with corresponding traits from the UK Biobank (**Supplementary Data 4**). For complex phenotypes, we compared them with all relevant components from the UK Biobank dataset, resulting in 34 pairwise comparisons. Out of these, 26 comparisons were nominally significant ($p < 0.05$), and 22 passed the Bonferroni significance threshold (0.05/34=0.00147).

From the FinnGen traits, we specifically selected dyslipidemia, hypertension, type 2 diabetes (T2D), obesity, myocardial infarction, ischemic heart disease, anxiety, depression, smoking, and sleep apnea. We then examined the genetic correlation between these chosen traits and their corresponding counterparts from our pool of 35 traits. We constructed 18 pairs and found nominal significance for 15 of them, while 8 passed the Bonferroni significance threshold (0.05/18=0.00278) (**Supplementary Data 4**).

Interestingly, we observed a significant negative correlation between the trait 'Snoring' in the Russian Biobank and the same trait in the UK Biobank (rg= -0.4491, p=0.0003). Simultaneously, the trait 'Snoring' from the Russian Biobank displayed a positive correlation with the trait 'Sleep apnea' from FinnGen (rg= 0.3106, p=0.0133). We further investigated and discovered a strong negative correlation between the trait 'Snoring' from the UK Biobank and the trait 'Sleep apnea' from FinnGen (rg= -0.5343, p<1.10-16). This observation suggests the possibility of a flip for the particular 'Snoring' GWAS in the UK Biobank.

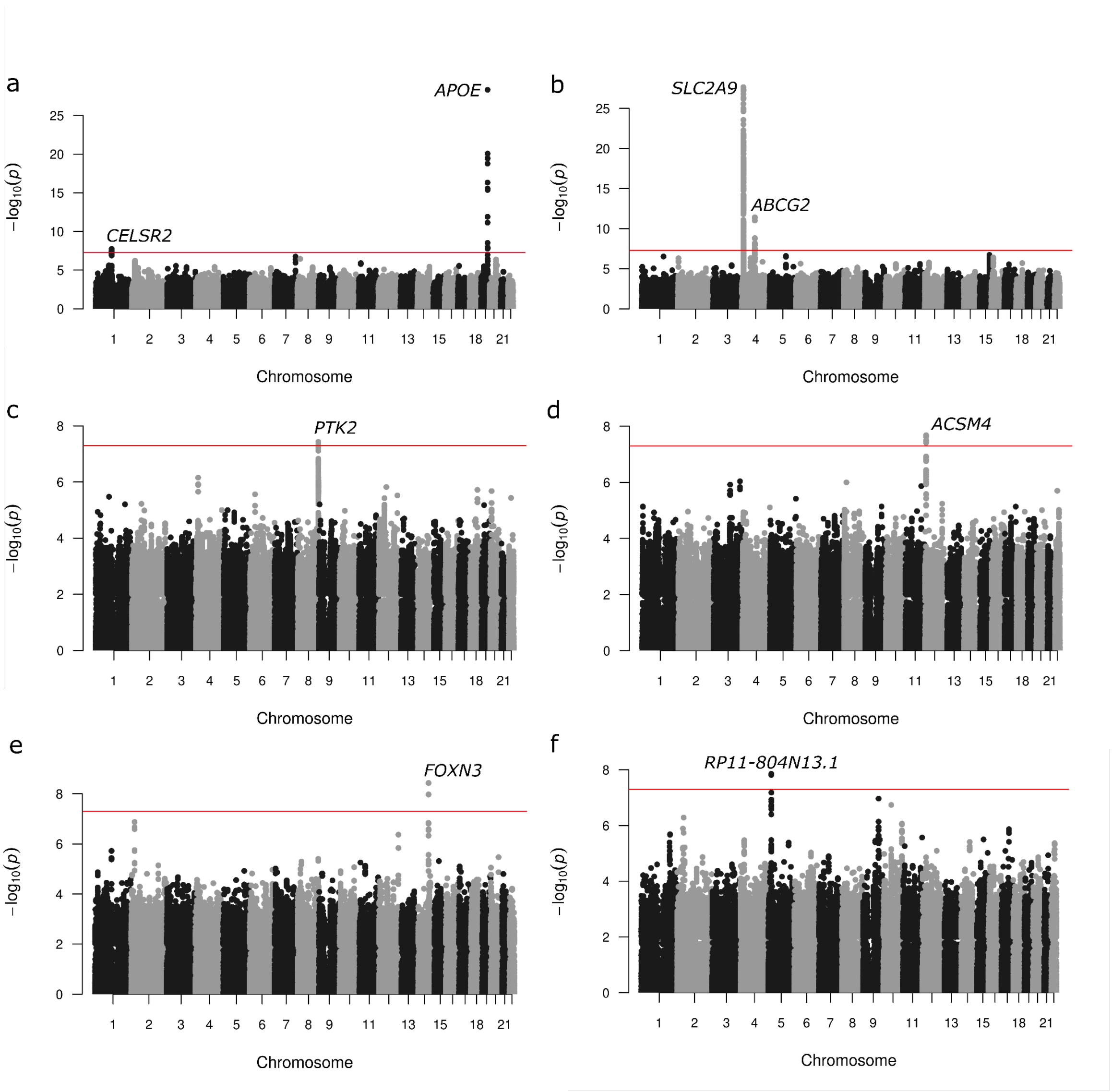

Supplementary Figure 24. GWAS. **a** LDL levels; **b** Uric acid levels; **c** Smoking (never); **d** Smoking (current); **e** Abdominal obesity; **f** Increased blood pressure in the second half of pregnancy

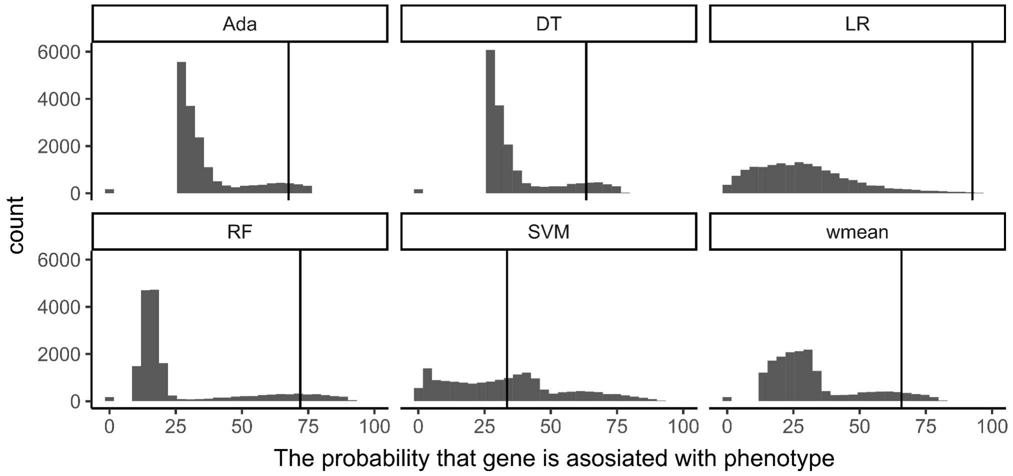

Supplementary Figure 25. The results of PTK2 gene prioritization with Never smoke phenotype. The black line is the value for *PKT2* gene

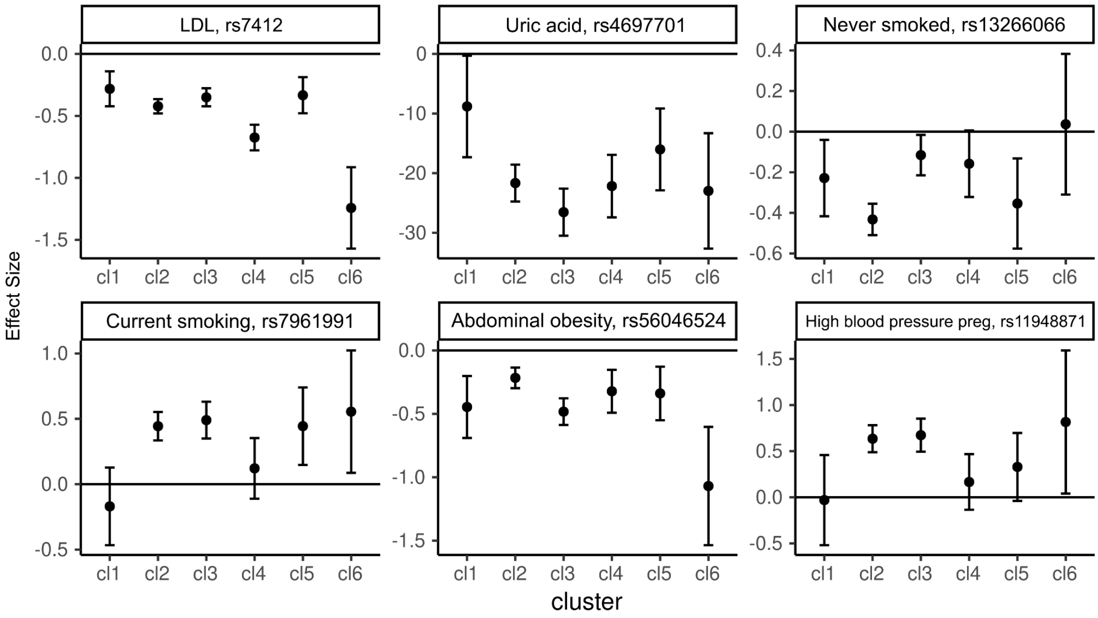

Supplementary Figure 26. Effect sizes with standard errors for described associations among different Russian clusters.

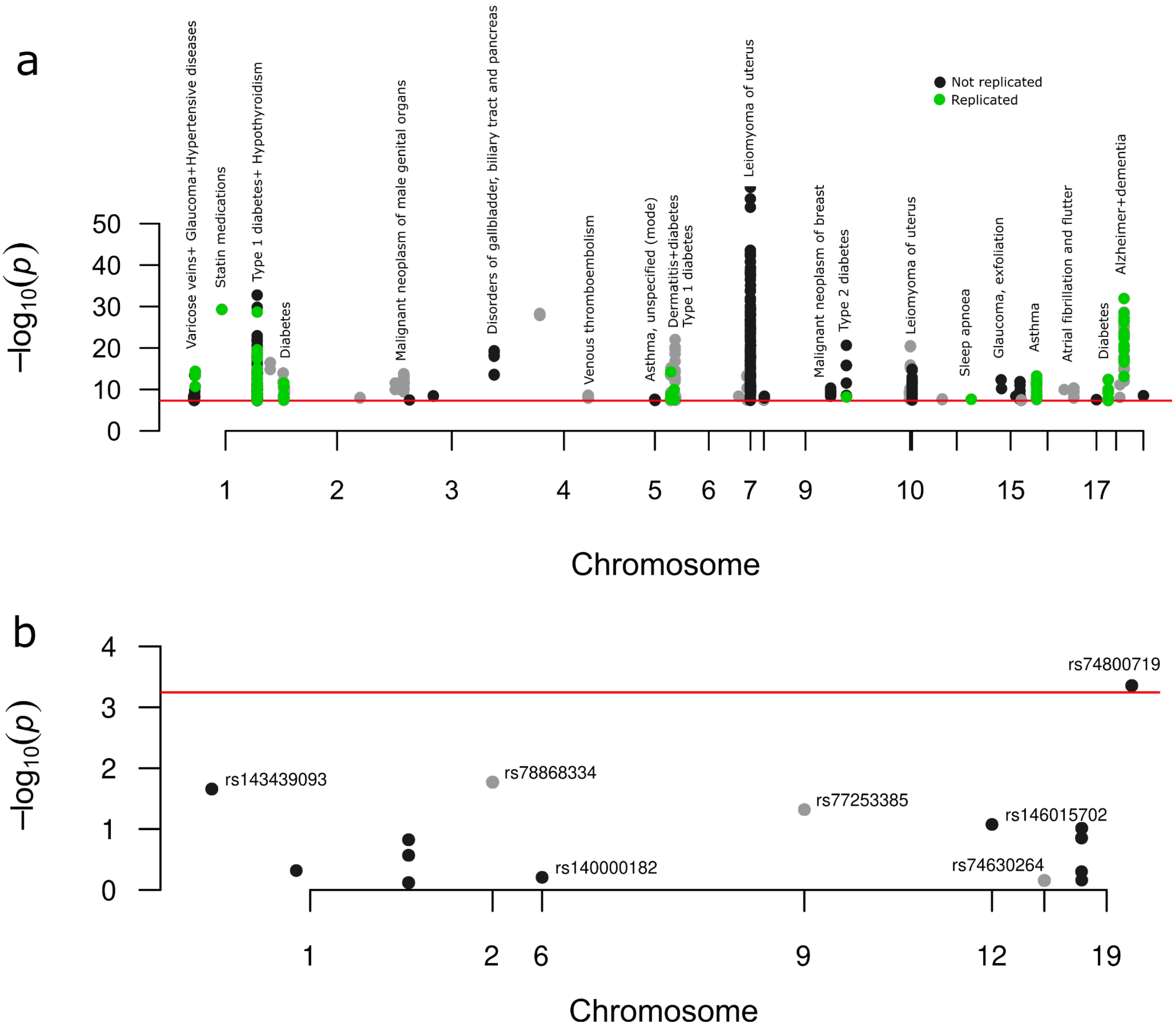

Supplementary Figure 27. Replication of genome-wide significant Finnish enriched variants (MAF RUS > 0.01, log-ratio EAS NFE < 2) in Russian biobank; **a** All genome-wide significant Finnish enriched variants (MAF RUS > 0.01, log-ratio EAS NFE < 2); **b** Replication of LD-clumped variants united in 8 phenotypes

References:

1. Simon, P. D. The 10-item Perceived Stress Scale as a valid measure of stress perception. *Asia Pac. Psychiatry* **13**, e12420 (2021).
2. Herrero, M. J. *et al.* A validation study of the hospital anxiety and depression scale (HADS) in a Spanish population. *Gen. Hosp. Psychiatry* **25**, 277–283 (2003).
3. Sorlie, P. D., Cooper, L., Schreiner, P. J., Rosamond, W. & Szklo, M. Repeatability and validity of the Rose questionnaire for angina pectoris in the Atherosclerosis Risk in Communities Study. *J. Clin. Epidemiol.* **49**, 719–725 (1996).
4. Oei, H.-H. S. *et al.* The association of Rose questionnaire angina pectoris and coronary calcification in a general population: the Rotterdam Coronary Calcification Study. *Ann. Epidemiol.* **14**, 431–436 (2004).
5. Alberti, K. G. M. M. *et al.* Harmonizing the metabolic syndrome: a joint interim statement of the International Diabetes Federation Task Force on Epidemiology and Prevention; National Heart, Lung, and Blood Institute; American Heart Association; World Heart Federation; International Atherosclerosis Society; and International Association for the Study of Obesity. *Circulation* **120**, 1640–1645 (2009).
6. Williams, B. *et al.* 2018 ESC/ESH Guidelines for the management of arterial hypertension: The Task Force for the management of arterial hypertension of the European Society of Cardiology and the European Society of Hypertension: The Task Force for the management of arterial hypertension of the European Society of Cardiology and the European Society of Hypertension. *J. Hypertens.* **36**, 1953–2041 (2018).
7. Vlachopoulos, C. *et al.* Association of Estimated Pulse Wave Velocity With Survival: A Secondary Analysis of SPRINT. *JAMA Netw Open* **2**, e1912831 (2019).

8. Mach, F. *et al.* 2019 ESC/EAS Guidelines for the management of dyslipidaemias: lipid modification to reduce cardiovascular risk. *Eur. Heart J.* **41**, 111–188 (2020).
9. Stevens, L. A. *et al.* Comparative performance of the CKD Epidemiology Collaboration (CKD-EPI) and the Modification of Diet in Renal Disease (MDRD) Study equations for estimating GFR levels above 60 mL/min/1.73 m2. *Am. J. Kidney Dis.* **56**, 486–495 (2010).
10. Authors/Task Force Members: *et al.* 2021 ESC Guidelines for the diagnosis and treatment of acute and chronic heart failure: Developed by the Task Force for the diagnosis and treatment of acute and chronic heart failure of the European Society of Cardiology (ESC). With the special contribution of the Heart Failure Association (HFA) of the ESC. *Eur. J. Heart Fail.* **24**, 4–131 (2022).
11. Song, K. *et al.* Prediction of Insulin Resistance by Modified Triglyceride Glucose Indices in Youth. *Life* **11**, (2021).
12. Janghorbani, M., Adineh, H. & Amini, M. Evaluation of the Finnish Diabetes Risk Score (FINDRISC) as a screening tool for the metabolic syndrome. *Rev. Diabet. Stud.* **10**, 283–292 (2013).
13. Meigs, J. B. *et al.* Body mass index, metabolic syndrome, and risk of type 2 diabetes or cardiovascular disease. *J. Clin. Endocrinol. Metab.* **91**, 2906–2912 (2006).
14. Pearson, T. A. *et al.* Markers of inflammation and cardiovascular disease: application to clinical and public health practice: A statement for healthcare professionals from the Centers for Disease Control and Prevention and the American Heart Association. *Circulation* **107**, 499–511 (2003).
15. Derwahl, K.-M., Duntas, L. H. & Butz, S. *The Thyroid and Cardiovascular Risk: Merck European Thyroid Symposium, Berlin 2004, June 10-13*. (2005).
16. Teixeira, P. F. S. *et al.* Treatment of subclinical hypothyroidism reduces atherogenic lipid levels in a placebo-controlled double-blind clinical trial. *Horm. Metab. Res.* **40**, 50–55 (2008).
17. Kronenberg, F. *et al.* Lipoprotein(a) in atherosclerotic cardiovascular disease and aortic

stenosis: a European Atherosclerosis Society consensus statement. *Eur. Heart J.* **43**, 3925–3946 (2022).

18. Stein, J. H. *et al.* Use of carotid ultrasound to identify subclinical vascular disease and evaluate cardiovascular disease risk: a consensus statement from the American Society of Echocardiography Carotid Intima-Media Thickness Task Force. Endorsed by the Society for Vascular Medicine. *J. Am. Soc. Echocardiogr.* **21**, 93–111; quiz 189–90 (2008).
19. Van Bortel, L. M. *et al.* Expert consensus document on the measurement of aortic stiffness in daily practice using carotid-femoral pulse wave velocity. *J. Hypertens.* **30**, 445–448 (2012).
20. Saiki, A. *et al.* New Horizons of Arterial Stiffness Developed Using Cardio-Ankle Vascular Index (CAVI). *J. Atheroscler. Thromb.* **27**, 732–748 (2020).
21. Dachun Xu *et al.* Sensitivity and specificity of the ankle--brachial index to diagnose peripheral artery disease: a structured review. *Vasc. Med.* **15**, 361–369 (2010).
22. D'Agostino, R. B., Sr *et al.* General cardiovascular risk profile for use in primary care: the Framingham Heart Study. *Circulation* **117**, 743–753 (2008).
23. Goff, D. C., Jr *et al.* 2013 ACC/AHA guideline on the assessment of cardiovascular risk: a report of the American College of Cardiology/American Heart Association Task Force on Practice Guidelines. *J. Am. Coll. Cardiol.* **63**, 2935–2959 (2014).
24. Conroy, R. M. *et al.* Estimation of ten-year risk of fatal cardiovascular disease in Europe: the SCORE project. *Eur. Heart J.* **24**, 987–1003 (2003).
25. Piepoli, M. F. *et al.* 2016 European Guidelines on cardiovascular disease prevention in clinical practice: The Sixth Joint Task Force of the European Society of Cardiology and Other Societies on Cardiovascular Disease Prevention in Clinical Practice (constituted by representatives of 10 societies and by invited experts)Developed with the special contribution of the European Association for Cardiovascular Prevention & Rehabilitation (EACPR). *Eur. Heart J.* **37**, 2315–2381 (2016).
26. Tillmann, T. *et al.* Development and validation of two SCORE-based cardiovascular risk

prediction models for Eastern Europe: a multicohort study. *Eur. Heart J.* **41**, 3325–3333 (2020).

27. Jdanov, D. A. *et al.* Recalibration of the SCORE risk chart for the Russian population. *Eur. J. Epidemiol.* **29**, 621–628 (2014).
28. Kurki, M. I. *et al.* FinnGen provides genetic insights from a well-phenotyped isolated population. *Nature* **613**, 508–518 (2023).
29. McCarthy, S. *et al.* A reference panel of 64,976 haplotypes for genotype imputation. *Nat. Genet.* **48**, 1279–1283 (2016).
30. Kolosov, N. *et al.* Genotype imputation and polygenic score estimation in northwestern Russian population. *PLoS One* **17**, e0269434 (2022).
31. Browning, B. L., Zhou, Y. & Browning, S. R. A One-Penny Imputed Genome from Next-Generation Reference Panels. *Am. J. Hum. Genet.* **103**, 338–348 (2018).
32. Chang, C. C. *et al.* Second-generation PLINK: rising to the challenge of larger and richer datasets. *Gigascience* **4**, 7 (2015).
33. The R Project for Statistical Computing. https://www.R-project.org/.
34. igraph – Network analysis software. https://igraph.org.
35. GitHub - hail-is/hail: Cloud-native genomic dataframes and batch computing. *GitHub* https://github.com/hail-is/hail.
36. adamethods: Archetypoid Algorithms and Anomaly Detection. *Comprehensive R Archive Network (CRAN)* https://CRAN.R-project.org/package=adamethods.
37. GitHub - alexloboda/SVDFunctions. *GitHub* https://github.com/alexloboda/SVDFunctions.
38. Alexander, D. H., Novembre, J. & Lange, K. Fast model-based estimation of ancestry in unrelated individuals. *Genome Res.* **19**, 1655–1664 (2009).
39. Danecek, P. *et al.* The variant call format and VCFtools. *Bioinformatics* **27**, 2156–2158 (2011).
40. Browning, S. R. & Browning, B. L. Rapid and accurate haplotype phasing and missing-data inference for whole-genome association studies by use of localized haplotype

clustering. *Am. J. Hum. Genet.* **81**, 1084–1097 (2007).

41. Various R Programming Tools for Plotting Data [R package gplots version 3.1.3]. (2022).
42. Hunter, J. D. Matplotlib: A 2D Graphics Environment. *Comput. Sci. Eng.* **9**, 90–95 (2007).
43. Browning, S. R. & Browning, B. L. Accurate Non-parametric Estimation of Recent Effective Population Size from Segments of Identity by Descent. *Am. J. Hum. Genet.* **97**, 404–418 (2015).
44. Pagani, L. *et al.* Genomic analyses inform on migration events during the peopling of Eurasia. *Nature* **538**, 238–242 (2016).
45. Bergström, A. *et al.* Insights into human genetic variation and population history from 929 diverse genomes. *Science* **367**, (2020).
46. Pickrell, J. K. & Pritchard, J. K. Inference of population splits and mixtures from genome-wide allele frequency data. *PLoS Genet.* **8**, e1002967 (2012).
47. Liu, M. *et al.* Association studies of up to 1.2 million individuals yield new insights into the genetic etiology of tobacco and alcohol use. *Nat. Genet.* **51**, 237–244 (2019).
48. Kolosov, N., Daly, M. J. & Artomov, M. Prioritization of disease genes from GWAS using ensemble-based positive-unlabeled learning. *Eur. J. Hum. Genet.* **29**, 1527–1535 (2021).
