# Supplementary material for: Complex trait susceptibilities and population diversity in a sample of 4,145 Russians
Source: Nat Commun. 2024 Jul 23;15:6212. doi: 10.1038/s41467-024-50304-1 (PMC11266540; doi:10.1038/s41467-024-50304-1)
Supplement: Supplementary file 3 — Description of Additional Supplementary Files [file 41467_2024_50304_MOESM3_ESM.pdf]

Description of Additional Supplementary Files

File Name: Supplementary Data 1

Description: Biannual updates of vital status of ESSE-RF cohort

File Name: Supplementary Data 2

Description: Enrichment of unique Finnish variants in Russian and East Asian populations

File Name: Supplementary Data 3

Description: FinnGen traits used to replicat unique Finnish variants in Russian Biobank

File Name: Supplementary Data 4

Description: Genetic correlations between phenotypes from Russian Biobank and from UK Biobank / FinnGen (phenotypes that passed the Bonferroni correction are shown in yellow)

File Name: Supplementary Data 5

Description: Trimmed with 5th and 95th quantiles phenotypes
