# Supplementary material for: Complex trait susceptibilities and population diversity in a sample of 4,145 Russians
Source: Nat Commun. 2024 Jul 23;15:6212. doi: 10.1038/s41467-024-50304-1 (PMC11266540; doi:10.1038/s41467-024-50304-1)
Supplement: Supplementary file 5 — Reporting Summary [file 41467_2024_50304_MOESM5_ESM.pdf]

nature portfolio

Corresponding author(s): Mykyta Artomov

Last updated by author(s): Mar 1, 2024

Reporting Summary

Nature Portfolio wishes to improve the reproducibility of the work that we publish. This form provides structure for consistency and transparency in reporting. For further information on Nature Portfolio policies, see our Editorial Policies and the Editorial Policy Checklist.

Statistics

For all statistical analyses, confirm that the following items are present in the figure legend, table legend, main text, or Methods section.

n/a	Confirmed	
☐	☒	The exact sample size (*n*) for each experimental group/condition, given as a discrete number and unit of measurement
☐	☒	A statement on whether measurements were taken from distinct samples or whether the same sample was measured repeatedly
☐	☒	The statistical test(s) used AND whether they are one- or two-sided<br>*Only common tests should be described solely by name; describe more complex techniques in the Methods section.*
☐	☒	A description of all covariates tested
☐	☒	A description of any assumptions or corrections, such as tests of normality and adjustment for multiple comparisons
☐	☒	A full description of the statistical parameters including central tendency (e.g. means) or other basic estimates (e.g. regression coefficient) AND variation (e.g. standard deviation) or associated estimates of uncertainty (e.g. confidence intervals)
☐	☒	For null hypothesis testing, the test statistic (e.g. *F*, *t*, *r*) with confidence intervals, effect sizes, degrees of freedom and *P* value noted<br>*Give P values as exact values whenever suitable.*
☒	☐	For Bayesian analysis, information on the choice of priors and Markov chain Monte Carlo settings
☒	☐	For hierarchical and complex designs, identification of the appropriate level for tests and full reporting of outcomes
☒	☐	Estimates of effect sizes (e.g. Cohen's *d*, Pearson's *r*), indicating how they were calculated

Our web collection on statistics for biologists contains articles on many of the points above.

Software and code

Policy information about availability of computer code

Data collection	No data collection software was used.
Data analysis	The GWAS results were annotated with VEP [33] and integrated into a PheWeb database [34]. We provide access through an online portal, Biobank Russia: https://biobank.almazovcentre.ru. Furthermore, genetic correlations were calculated between all pairs of phenotypes using LD-score regression, and significant values are shown in the PheWeb database [35,36]. Additionally, LD-score regression was used to calculate heritability of phenotypes from Biobank Russia and genetic correlation between corresponding phenotypes from UK Biobank and FinnGen. We used POSTGAP[37] and GPrior [38] for gene mapping and prioritization in GWAS to confirm our findings in a case study of smoking status phenotypes. The R 'ieugwasr' (v0.1.5) was used to retrieve FinnGen PheWas using the batch 'finn-b' [39] for replication. R 'TwoSampleMR' (v0.5.6) was used to clump genome-wide significant Finnish enriched variants [40].<br>Original dataset of 823 phenotypes was subjected to a quality filtration prior to GWAS using R-libraries: 'dplyr' (v1.0.0) and 'tidyr' (v1.1.1)<br>Custom codes used in the manuscript can be found at: https://github.com/ArtomovLab/RUS_BB/

For manuscripts utilizing custom algorithms or software that are central to the research but not yet described in published literature, software must be made available to editors and reviewers. We strongly encourage code deposition in a community repository (e.g. GitHub). See the Nature Portfolio guidelines for submitting code & software for further information.

Data

Policy information about availability of data

All manuscripts must include a data availability statement. This statement should provide the following information, where applicable:

- Accession codes, unique identifiers, or web links for publicly available datasets
- A description of any restrictions on data availability
- For clinical datasets or third party data, please ensure that the statement adheres to our policy

All GWAS, PheWAS, allele frequencies, and aggregated data, along with visualization, are openly available on the Biobank Russia portal: https://biobank.almazovcentre.ru

Research involving human participants, their data, or biological material

Policy information about studies with human participants or human data. See also policy information about sex, gender (identity/presentation), and sexual orientation and race, ethnicity and racism.

Reporting on sex and gender	Only summary information about the dataset is provided with respect to sex and gender distribution. No sex-specific analyses were carried out.
Reporting on race, ethnicity, or other socially relevant groupings	We do not use discrete labels for the ancestry groups. Instead we identify relatively homogeneous population clusters using PCA and characterize them through the haplotype structure analysis, which provides quantitative description of the ancestral composition.
Population characteristics	We use the cohort collected through a random sampling of several metro areas. Cohort includes all populations found within sampled territories. Population mean age was 46+/-12 (SD) years. Range 18-65. Genotypic information was obtained from all participants
Recruitment	A cohort of 4,800 residents of three areas in Russia – St. Petersburg (N=1,600), Orenburg (N=1,600) and Samara (N=1,600) were recruited in 2012-2013 through an ambulatory visit to local hospitals and polyclinics.<br>Each patient was invited for an ambulatory visit for one day to collect phenotypic information.<br>For the St. Petersburg cohort, several additional phenotypes were collected: measurements of blood pressure and heart rate in standing position, vessel stiffness measurements and electrocardiogram (ECG), urine albumin and extended blood metabolic panel test with additional test for C-reactive protein (CRP), lipoprotein (a), apolipoproteins A and B, cortisol, leptin, adiponectin, and vitamin D.<br>In 2018-2019, 289 out of 1,600 original patients from St. Petersburg were invited for an additional ambulatory visit as a part of different local studies (familial hypercholesterolemia, metabolically healthy obesity, premature vascular aging).
Ethics oversight	local ethics committees (Almazov National Medical Research Center, St. Petersburg), the institutional review board of Massachusetts General Hospital (IRB #2014P000459) and the Nationwide Children's Hospital (IRB #00002944)

Note that full information on the approval of the study protocol must also be provided in the manuscript.

Field-specific reporting

Please select the one below that is the best fit for your research. If you are not sure, read the appropriate sections before making your selection.

☒ Life sciences ☐ Behavioural & social sciences ☐ Ecological, evolutionary & environmental sciences

For a reference copy of the document with all sections, see nature.com/documents/nr-reporting-summary-flat.pdf

Life sciences study design

All studies must disclose on these points even when the disclosure is negative.

Sample size	The cohort included equal number of participants (1,600) randomly sampled in 3 metro areas. Additionally, we included legacy cohorts that existed as sample collections prior to 2020. We have not carried out the sample size calculations, as this is the largest clinically-based biobank data available in Russia to date.
Data exclusions	Duplicated samples, ancestral outliers, samples with poor quality of genotyping, related individuals were excluded from the analysis.
Replication	Previously known associations from UK biobank were replicated in our data as a positive control. 2 novel associations in our data were also replicated in the UK biobank. Finnish-specific association of Alzheimer disease was replicated in our data. Other, unique to our data associations were not found in other publicly available datasets.
Randomization	The study does not involve intervention, therefore, no randomization was performed. The participants of the study were recruited at random in the corresponding metro-areas.
Blinding	The study does not involve intervention, therefore, no blinding was performed.

Reporting for specific materials, systems and methods

We require information from authors about some types of materials, experimental systems and methods used in many studies. Here, indicate whether each material, system or method listed is relevant to your study. If you are not sure if a list item applies to your research, read the appropriate section before selecting a response.

Materials & experimental systems

n/a	Involved in the study	
☒	☐	Antibodies
☒	☐	Eukaryotic cell lines
☒	☐	Palaeontology and archaeology
☒	☐	Animals and other organisms
☒	☐	Clinical data
☒	☐	Dual use research of concern
☒	☐	Plants

Methods

n/a	Involved in the study	
☒	☐	ChIP-seq
☒	☐	Flow cytometry
☒	☐	MRI-based neuroimaging
